# Decreasing admissions in older and increasing mortality in younger patients: a nationwide observational study of all German ICU cases 2011–2022

**DOI:** 10.1186/s13054-026-05924-y

**Published:** 2026-03-06

**Authors:** Sarah-Yasmin Thomsen, Nassim Kakavand, Friedrich Alexander von Samson-Himmelstjerna, Ingo Eitel, Christian Jung, Roland Schmitt, Kevin Schulte, Benedikt Kolbrink

**Affiliations:** 1 Department of Nephrology and Hypertension, University Hospital Schleswig-Holstein Kiel, Christian-Albrechts University, Arnold-Heller-Str. 3, 24105, Kiel, Germany; 2 Department of Anaesthesiology and Operative Intensive Care Medicine, University Hospital Schleswig-Holstein Kiel, Christian-Albrechts University, Kiel, Germany; 3 Department of Cardiology, Angiology and Intensive Care Medicine, University Hospital Schleswig-Holstein LübeckUniversity of Lübeck, Lübeck, Germany; 4 Division of Cardiology, Pulmonary Diseases, Vascular Medicine, University Hospital Düsseldorf, Medical Faculty, University, Düsseldorf, Germany

**Keywords:** Intensive care unit, Aging population, Mortality, Organ replacement therapy, Health services research, Epidemiology

## Abstract

**Background:**

Demographic aging is expected to substantially affect intensive care medicine, with a growing proportion of elderly patients and increasing clinical complexity, while resources remain limited. It is therefore critical for clinicians and health systems to understand, how intensive care unit (ICU) admissions, outcomes, and treatment intensity across patients of differing age have evolved in recent years. Longitudinal data on these parameters are scarce on the population level.

**Methods:**

We conducted a retrospective population-based analysis of all German inpatient cases from 2011–2022. Hospitalizations with ICU treatment ≥ 24 h were included. Temporal trends in ICU admissions, in-hospital mortality, ICU-related deaths, and use of organ replacement therapies (ORT) were analyzed and stratified by age groups. ORTs comprised mechanical ventilation, renal replacement therapy, and extracorporeal membrane oxygenation.

**Results:**

Overall, 8.4 million ICU admissions were analyzed. During the observation period, ICU admissions showed an overall decline of 12.6%, driven by reductions in patients aged 65–79 years (− 18.1%) and ≥ 80 years (− 24%), while remaining stable in younger patients. Despite fewer admissions, ICU-related mortality showed an overall increase from 14.4% to 18.9%, mainly in patients < 80 years. Overall, in-hospital deaths declined across all age groups, but the proportion of ICU-related deaths rose among patients < 80 years. ORT use increased across all age groups and was associated with persistently high mortality, particularly with combined ORTs (up to 79%). Patients aged 65–79 years received combined ORTs most frequently. Patients ≥ 80 years had the highest mortality.

**Conclusions:**

ICU utilization and outcomes in Germany have shifted markedly over the past decade. Declining admissions among older patients, increasing ICU-related mortality in younger patients, and rising treatment intensity underscore age-specific differences in critical care delivery and outcomes, with important implications for ICU admission practices, treatment decisions and future resource planning.

**Supplementary Information:**

The online version contains supplementary material available at 10.1186/s13054-026-05924-y.

## Background

Treatment in intensive care units (ICUs) provides maximal care for critically ill patients and remains essential for achieving favourable outcomes, even in life-threatening situations. The ongoing demographic shift toward aging societies has led to a growing demand for intensive care among elderly patients in many industrialized countries [1].

Higher age is associated with a greater prevalence of severe illness, increased physiological instability, and a higher burden of comorbidities [2, 3]. These developments have significant implications for healthcare systems, exacerbating existing shortages of medical personnel and creating multiple challenges for individualized treatment [3–5].

In critically ill patients, particularly those admitted to ICUs, highly invasive organ replacement therapies (ORTs) often represent the final therapeutic option. Despite predictably poor outcomes in this population [2, 3, 6], elderly and very elderly patients regularly receive ORTs such as mechanical ventilation (VT), extracorporeal membrane oxygenation (ECMO), and renal replacement therapy (RRT) [7–9].

Due to the aging populations, it could therefore be assumed that we will treat more elderly patients with more invasive ORTs in ICUs. Consequently, the overall outcomes in ICUs would be expected to deteriorate. However, this assumption contrasts with reports describing substantial improvements in ICU outcomes despite increasing disease burden and severity [10, 11].

Although this question is highly relevant for both medicine and health policy in industrialized countries, detailed recent data on admission trends, mortality, and treatment practices for elderly and very elderly ICU patients remain scarce. The aim of this study was therefore to assess temporal trends in age-specific in-hospital mortality of patients admitted to the ICU, as well as the association between use of ORTs and mortality over time in Germany, the largest Central European country.

## Methods

### Data set

We performed a retrospective analysis of anonymized nationwide data from the German Federal Statistical Office (GFSO) based on diagnosis-related groups (DRG) statistics as described previously [12, 13]. This administrative dataset, collected originally for reimbursement purposes but widely used in health services research, includes all inpatient cases in Germany with information on demographics, diagnoses, procedures, and discharge status. Diagnoses were coded using the International Classification of Diseases, 10th Revision, German Modification (ICD-10-GM), and procedures using the German Operation and Procedure Classification System (*Operationen- und Prozedurenschlüssel,* OPS), the national coding standard for medical procedures and interventions.

### Study period and inclusion criteria

We included all patient cases with OPS codes indicating ICU treatment for ≥ 24 h between 2011 and 2022, and ORT with VT (invasive, non-invasive, continuous positive airway pressure (CPAP)), RRT (intermittent, continuous, prolonged, or peritoneal dialysis), and ECMO (veno-venous and veno-arterial, also referred to as extracorporeal life support). An overview of all ICD- and OPS-codes used in the analyses is available in supplementary tables 1/2. Age groups were categorized as follows: younger patients (< 65 years), elderly patients (65–79 years), and very elderly patients (≥ 80 years), using a pragmatic, literature-consistent stratification for population-level analyses [14, 15].

### Endpoints

The primary endpoint was all-cause, in-hospital mortality of ICU patients. “ICU patient” was defined as any hospitalized patient with at least one ICU admission during hospital stay. For clarity and consistency, all cause, in-hospital mortality of ICU patients and all cause, in-hospital deaths of ICU patients will be referred to as "ICU-related mortality" and "ICU-related deaths" throughout the text. For calculation of sex- and age-specific incidence of ICU and hospital admissions as well as deaths in relation to the German general population, we used the databank GENESIS-online by the GFSO (https://www-genesis.destatis.de/datenbank/online/statistic/12411/details).

### Exclusion criteria

For each item queried, we checked the data set for completeness and excluded patient cases with missing information. Of all patient cases in the data set, 323 (0.004%) were excluded due to missing information about sex.

### Statistical analysis

To query the database, we created syntax files using R (version 4.1.2) (Supplementary Table 3). Statistical analyses were conducted using GraphPad Prism (version 10.4.1) and R (version 4.1.2).

To evaluate temporal changes over the observation period, univariable linear regression models were fitted with calendar year as the independent variable and the respective annual outcome measure as the dependent variable. Annual outcome measures included ICU admissions and in-hospital mortality among ICU patients, analysed overall and stratified by age group and ORT. The average annual change (slope) over time was reported. Statistical significance of temporal trends was assessed using an F-test to determine whether the regression slope differed significantly from zero. Relative changes are reported as percentages. Model assumptions were assessed graphically. Linearity, the presence of outliers, and homoscedasticity were evaluated by visual inspection of time-series plots. Given the aggregated, annual nature of the data, observations were considered independent.

To evaluate the association of age and ORT with in-hospital mortality among ICU patients, univariable and multivariable binary logistic regression models were applied. The dependent variable was in-hospital death. Independent variables included age group (< 65 years, 65–79 years, ≥ 80 years) and the use of ORT categorized as no ORT, VT, RRT, ECMO and the combinations thereof.

Multivariable models included all covariables simultaneously. Independence of observations was assumed given the case-based structure of the dataset. Given the categorical and conceptually distinct predictors, relevant collinearity was considered unlikely and no indications of model instability were observed. Autocorrelation was not applicable, as the dataset did not include repeated measurements. Given the large sample size (n = 8,357,412) and the limited number of model parameters (k = 9), the risk of overfitting was considered negligible. Odds ratios (ORs) with corresponding 95% confidence intervals are reported. All *p*-values < *0.05* were considered statistically significant.

## Results

### Declining ICU and hospital admissions in Germany between 2011 and 2022

**Fig. 1:**
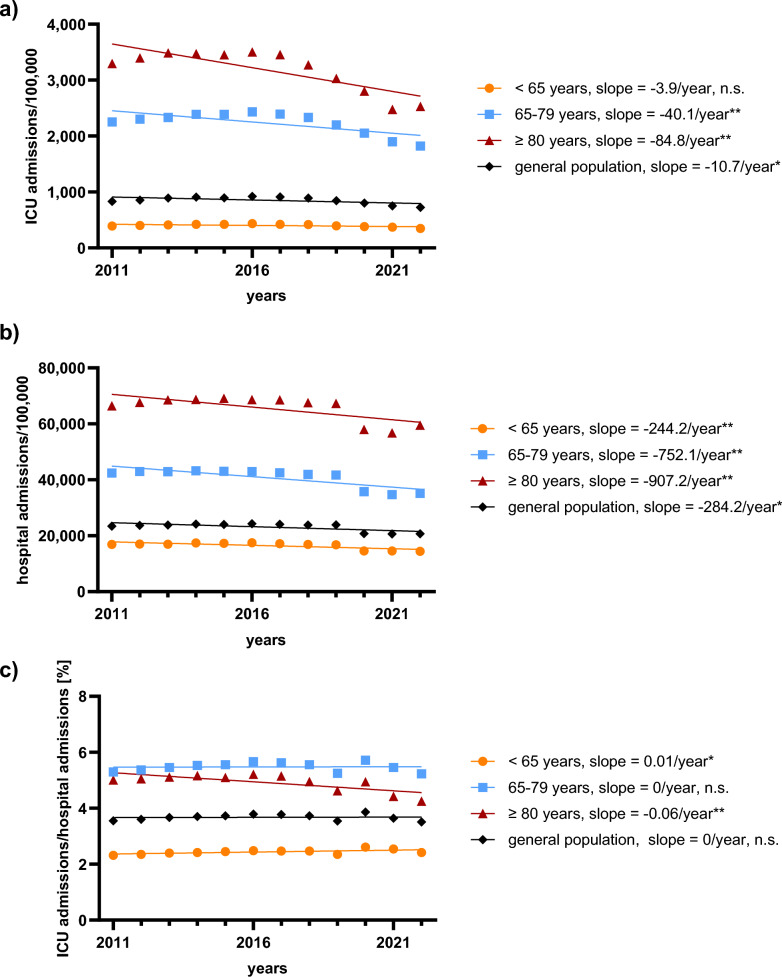
Incidence of ICU and hospital admissions in Germany, 2011–2022. (**a**) shows intensive care unit (ICU) admissions per 100,000 inhabitants, overall and stratified by age group. (**b**) displays hospital admissions per 100,000 inhabitants, overall and stratified by age group. (**c**) presents changes in the proportion of ICU admissions relative to hospital admissions over the observation period, overall and stratified by age group. Significance levels: * = p < 0.05; ** = p < 0.01; *** = p < 0.001; n.s. = not significant (p ≥ 0.05)

We analyzed 8,399,479 ICU admissions between 2011 and 2022 (primary diagnoses and causes of death are shown in the Supplementary Tables 4/5). During the observation period, ICU admissions normalized to the German population showed an overall decline of 12.6%, reflecting the direction and magnitude of the observed annual trends (from 830.9 to 726.4 per 100,000; *p* = 0.04). This decrease was observed in both elderly and very elderly patients, while ICU admissions among younger patients did not change significantly (younger: -10.6%, *p* = 0.07; elderly: − 18.1%, *p* = 0.008; very elderly: − 24.0%, *p* = 0.003) (Fig. 1a; Supplementary Table 6).

Similarly, the incidence of hospital admissions overall decreased by 11.5% (*p* = 0.01) (Fig. 1b; Supplementary Table 6) and consistently across all age groups (younger: − 14.3%, *p* = 0.005; elderly: − 17.1%, *p* = 0.002; very elderly: -10.4%, *p* = 0.009) (Fig. 1b; Supplementary Table 6). To account for overall changes in hospitalizations, ICU admissions were additionally analyzed relative to total hospital admissions, stratified by age group. The proportion of ICU admissions relative to hospital admissions slightly increased in younger patients and remained stable in elderly, while decreasing in very elderly patients (younger: + 4.3%, *p* = 0.04; elderly: -1.3%, *p* = 0.92; very elderly: − 15.2%, *p* = 0.006) (Fig. 1c; Supplementary Table 6).

**Fig. 2:**
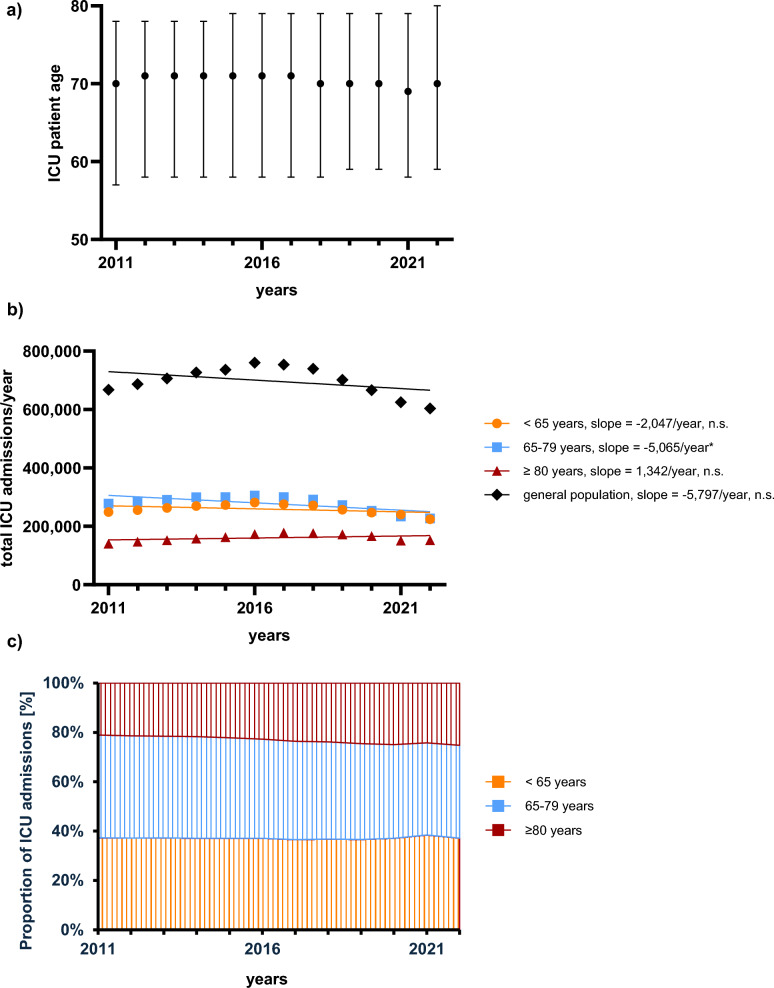
Age distribution of patients in German ICUs (2011–2022). (**a**) displays the median age of ICU patients (± IQR) over the study period, showing a stable median age of approximately 70 years. (**b**) presents the total number of admissions to German ICUs during the study period, both for the general population and stratified by age group. (**c**) shows the relative contribution to ICU admissions of age groups, with more than 60% of ICU patients being older than 65 years. Significance levels: * = p < 0.05; ** = p < 0.01; *** = p < 0.001; n.s. = not significant (p ≥ 0.05)

Although absolute ICU admissions and median patient age (70 years) remained stable (Fig. 2a/b) during the observation period, the proportion of ICU admissions accounted for by very elderly patients increased (21% to 25.2%, relative increase of + 19.4%, *p* < 0.001) (Fig. 2c). Patients > 65 years accounted for over 60% of ICU admissions throughout the study period (Fig. 2c). Population-adjusted ICU admissions among patients < 18 years of age did not show a significant temporal trend during the observation period (Supplementary Table 6).

### Increasing ICU-related mortality despite declining admissions

**Fig. 3:**
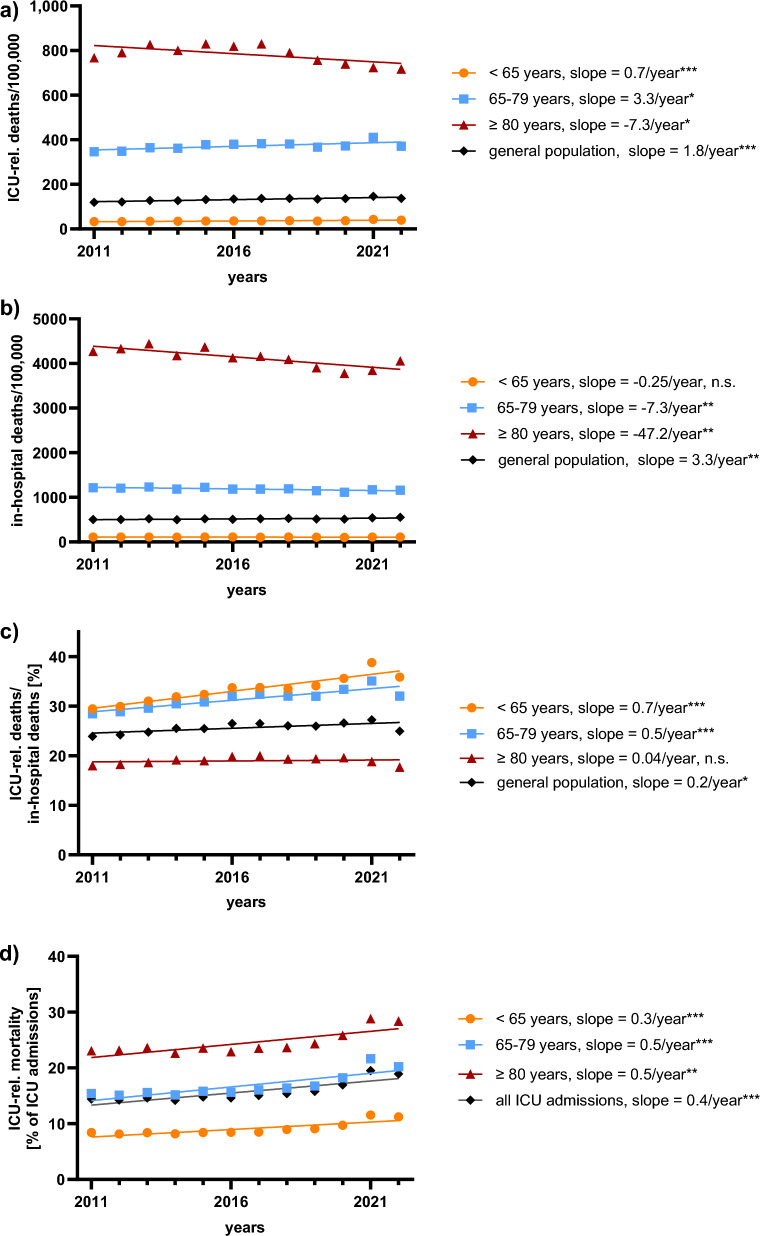
Deaths in German ICUs and hospitals, 2011–2022. (**a**) shows intensive care unit (ICU) deaths per 100,000 inhabitants, overall and stratified by age group. (**b**) displays in-hospital deaths per 100,000 inhabitants, overall and stratified by age group. (**c**) presents changes in the proportion of ICU-related deaths relative to in-hospital deaths over the observation period, overall and stratified by age group. (**d**) shows the trends in ICU-related mortality, overall and stratified by age group during the observation period. Significance levels: * = p < 0.05; ** = p < 0.01; *** = p < 0.001; n.s. = not significant (p ≥ 0.05)

The number of ICU-related deaths per 100,000 German inhabitants showed an overall increase of 14.8% over the observation period (from 119.7 to 137.4; *p* < 0.001) (Fig. 3a; Supplementary Table 7), but inconsistently across the different age groups. In patients < 80 years of age ICU-related deaths increased, but declined in very elderly patients (younger: + 19.2%, *p* < 0.001; elderly: + 7.4%, *p* = 0.013; very elderly: − 6.5%, *p* = 0.025) (Fig. 3a; Supplementary Table 7).

The number of all in-hospital deaths per 100,00 German inhabitants decreased among elderly and very elderly patients, while remaining stable among younger patients (younger: -2.2%, *p* = 0.26; elderly: − 4.7%, *p* = 0.003; very elderly: -4.9%, *p* = 0.001) (Fig. 3b; Supplementary Table 7). The proportion of ICU-related deaths among in-hospital deaths increased in patients < 80 years of age but remained stable in very elderly patients (younger: 29.5% to 35.9%, relative increase of + 21.8%, *p* < 0.001; elderly: 28.4% to 32%, relative increase of + 12.6%, *p* < 0.001; very elderly: 18% to 17.7%, relative decrease of − 1.7%, *p* = 0.6) (Fig. 3c; Supplementary Table 7). Furthermore, the number of both ICU and in-hospital deaths relative to hospital admissions significantly increased in patients < 80 years of age, whereas they stayed stable in very elderly patients (Supplementary Table 7 and Supplementary Fig. 1a/b).

During the observation period, the proportion of patients dying during hospitalizations with ICU-stay in Germany increased among those < 80 years of age, while remaining stable overall and in very elderly patients (younger: 15.1% to 17.6%, relative increase of + 16.6%, *p* < 0.001; elderly: 15.7% to 16.4%, relative increase of + 4.5%, *p* = 0.009; very elderly: 7.4% to 6.7%, relative decrease of -9.5%, *p* = 0.32) (Supplementary Table 7 and Supplementary Fig. 2a). In parallel, the proportion of all deaths occurring in hospitals in Germany declined across all age groups (Supplementary Table 7 and Supplementary Fig. 2b).

Throughout the study period, the incidence of ICU admissions was consistently higher in males than in females across all age groups. Mortality among ICU patients was highest in older male individuals (Supplementary Fig. 3a/b and Supplementary Table 8). Across the observation period, ICU-related deaths accounted for 13.9% of all deaths among men in the German population and 9.5% among women (Supplementary Fig. 3c and Supplementary Table 8).

### Increasing ORT use with persistently high mortality across all age groups

**Fig. 4:**
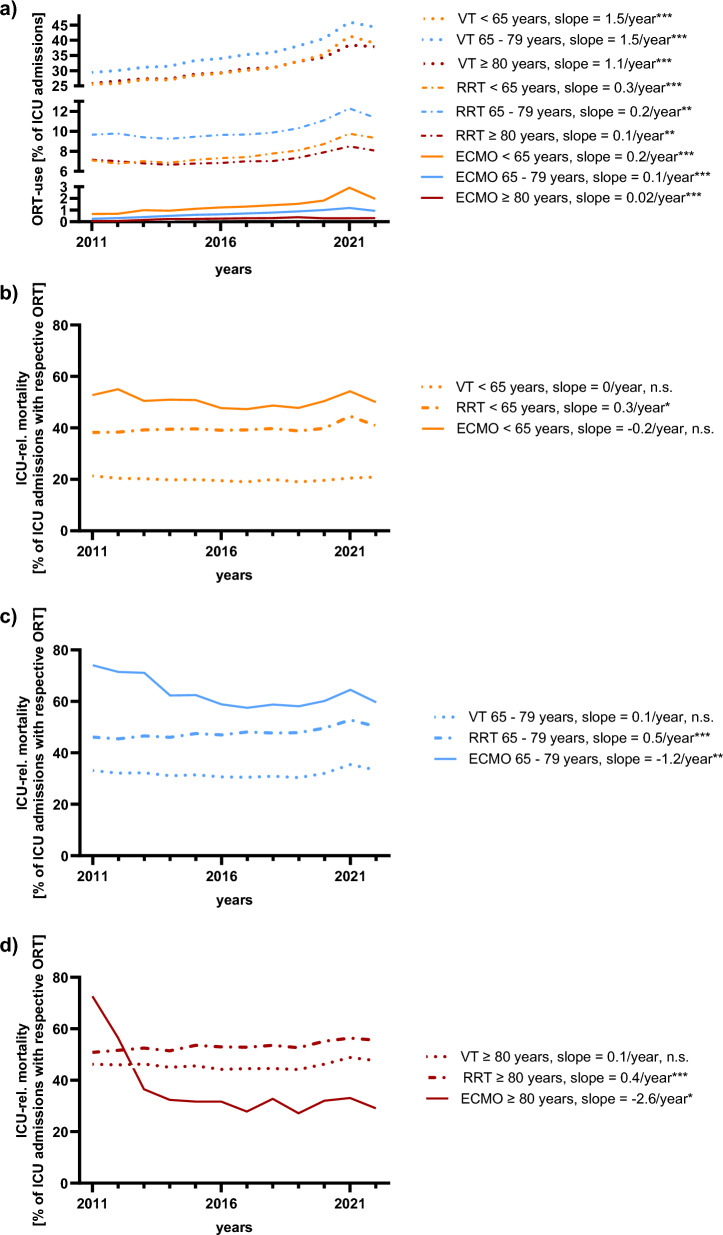
Overall mortality, and incidence and mortality of organ replacement therapies in German ICUs, 2011–2022 . (**a**) displays trends in the use of different organ replacement therapies (ORTs) over the observation period, stratified by age groups. Changes in ORT-related mortality over time, stratified by age group, are shown in panels (**b**) (< 65 years), (**c**) (65–79 years), and (**d**) (≥ 80 years). VT = mechanical ventilation, RRT = renal replacement therapy, ECMO = extracorporeal membrane oxygenation. Significance levels: * = p < 0.05; ** = p < 0.01; *** = p < 0.001; n.s. = not significant (p ≥ 0.05)

During the observation period, ICU-related mortality increased across all age groups, with the highest rate in very elderly patients (2011: 23.1%, 2022: 28.4%) (Fig. 3b; Supplementary Table 7). Similarly, the use of ORTs increased for all forms of ORT and across all age groups. Younger and very elderly patients showed similar rates for the use of VT and RRT, while elderly patients had the highest rates for both (Fig. 4a; Supplementary Table 9).

ORT-related mortality patterns varied: RRT mortality increased across all age groups (younger: 38.2% to 40.9%, relative increase of + 7.0%, *p* = 0.02; elderly: 46.1% to 50.2%, relative increase of + 9.0%, *p* < 0.001; very elderly: 50.9% to 55.5%, relative increase of + 9.2%, *p* = 0.008); VT mortality remained stable; ECMO mortality declined in elderly and very elderly but remained stable in younger patients (younger: 52.8% to 50.1%, relative decrease of -5.1%, *p* = 0.34; elderly: 74% to 59.6%, relative decrease of − 19.5%, *p* < 0.001; very elderly: 72.6% to 29.1%, relative decrease of − 59.9%, *p* = 0.009) (Fig. 4b-d; Supplementary Table 9).

Age-specific ORT incidence per 100,000 inhabitants was lowest for younger and highest for very elderly patients both for single ORT use, whereas combination of more than one ORT was less common in very elderly patients (Supplementary Fig. 4a/b, Supplementary Tables 10/11).

Average mortality for single ORTs ranged from 14–43% for VT and 21–36% for RRT. Combined ORTs were associated with substantially higher mortality, particularly for triple therapy (RRT + VT: 48–67%; VT + ECMO: 33–51%; RRT + ECMO + VT: 63–79%) (Supplementary Fig. 4c).

Univariable logistic regression analyses showed a strong association between ORT use and risk of death (Supplementary Table 11). Multivariable logistic regression analyses confirmed increased risk of death for all ORTs: VT or RRT alone yielded ~ fivefold increased risk of death; combined therapies yielded even higher risks, with 44-fold increased risk of death for triple therapy (Supplementary Table 11). Age was also independently associated with risk of death: when admitted to an ICU, elderly patients had a twofold, very elderly patients a threefold increased risk of death compared to younger patients (Supplementary Table 11).

## Discussion

Our population-based analysis of 8.4 million ICU admissions in Germany revealed three key findings: First, the incidence of ICU admissions declined over the past decade in Germany, particularly among elderly (65–79 years) and very elderly (≥ 80 years) patients. Second, ICU-related mortality increased, most notably among younger (< 65 years) patients. Third, we observed increasing use of ORTs in German ICU patients with poor outcomes and consistently high mortality across all age groups.

Earlier German data reported rising ICU admissions until 2015 [16]. Our findings reveal a reversal thereafter, with declining admission rates among elderly and very elderly patients—contrasting with the expected increase from demographic aging and the growing burden of critical illness and comorbidities [17, 18]. Similar trends have been observed in Canada [19], whereas other countries such as the Netherlands, Australia, and New Zealand report continuous growth in ICU admissions among older patients [6, 20].

Our observation in Germany may reflect several underlying mechanisms that cannot be directly disentangled in our data, among which three appear particularly plausible: (1) a genuine reduction in demand for ICU treatment in elderly, (2) potentially more selective admission practices, or (3) growing resource constraints. Regarding the first, absolute ICU admissions in very elderly patients remained stable, suggesting that the decline in rates more likely reflects changes in admission practice than a true reduction in need. Concerning the second, growing awareness of geriatric-specific issues—such as delirium, cognitive decline, and functional deterioration—has increasingly shaped clinical and political debates, which may have influenced admission decision-making, particularly under conditions of limited nursing staff [21, 22]. Moreover, the expansion of intermediate care units (IMCs) since the mid-2000s following recommendations by the German Interdisciplinary Association for Intensive and Emergency Medicine [23], may have redirected patients who previously would have received ICU treatment. Although this hypothesis is plausible, our data cannot directly confirm it. Finally, resource scarcity, particularly in the context of mandatory nurse staffing regulations introduced in Germany in 2019 (“*Pflegepersonaluntergrenzen*”) [24], represents an important health-system context that may have influenced ICU capacity, even before their formal implementation, when bed closures due to staffing shortages were already reported [4]. While our data cannot directly assess staffing effects, these factors are relevant for interpreting national ICU admission trends. Nevertheless, patients aged over 65 years continued to account for more than 60% of ICU admissions, underscoring the ongoing importance of providing optimal ICU care for older adults and recognizing their specific needs.

Despite decreasing ICU admissions in elderly and very elderly patients, overall mortality did not decline. Instead, the proportion of patients < 80 years dying during hospitalization with ICU treatment increased. This counterintuitive finding may have several explanations. First, Germany’s traditionally high ICU bed availability could have fostered overtreatment, including admissions of patients with advanced end-stage disease or very high severity at presentation, in whom survival chances are inherently limited [25–27]. Second, and in contrast, capacity pressures from staffing shortages and resulting bed closures may have produced a selection effect, with ICUs increasingly prioritizing patients with higher acute illness severity, thereby driving higher mortality. Under this hypothesis, a mortality decline might have been expected after the introduction of mandatory nurse staffing regulations, given the improved nurse-to-patient ratios. However, no such decrease was observed, possibly due to external confounders such as the COVID-19 pandemic, which may have influenced mortality trends in ways our data cannot fully disentangle. Third, worsening treatment quality must be considered: although staffing regulations aimed to improve safety and workload [24], shortages had already led to frequent ICU bed closures years before [4]. Even with formally improved ratios, overall resource scarcity, organizational adaptations, and increasing treatment complexity may have compromised care. Taken together, these findings highlight a complex interplay of demographic change, admission practices, and resource constraints. In line with the administrative nature of the data, the observed patterns are hypothesis-generating. Further research is urgently needed to disentangle these mechanisms and to guide strategies that ensure equitable, high-quality ICU care for an aging population.

We further observed a marked increase in ORT use over time, consistent with rising mortality rates. ICU admissions declined disproportionately among elderly and very elderly patients, with the latter rarely receiving combined ORTs. Elderly patients, however, still frequently received such therapies despite comparably poor outcomes. Mortality with ORT combinations, particularly RRT and ECMO, remained high (in our cohort 50–75%) [9, 28]. Interestingly, ECMO mortality decreased in very elderly patients while absolute numbers rose, likely reflecting stricter selection for robust candidates. These findings may indicate emerging age-driven selection, shaped by frailty, clinical decision-making, or growing acceptance of palliative care [16, 29]. Yet, a genuine change in therapeutic mindset in older patients would entail fewer ICU admissions, less ORT use, and lower mortality. The current discrepancy—declining admissions but continued aggressive treatment—may represent an early stage of shifting attitudes toward the needs and limits of intensive care in older adults.

Several limitations must be acknowledged. As a retrospective analysis of secondary data not originally intended for research purposes, post-discharge outcomes could not be assessed, and case-level validation was not possible. As a population-based analysis of administrative data, this study was designed to describe national trends in ICU utilization and outcomes rather than to provide fully risk-adjusted mortality estimates. Accordingly, residual confounding by unmeasured clinical factors such as frailty cannot be excluded, and disease severity was not modeled in detail. External factors, including the COVID-19 pandemic and temporal changes in administrative coding practices, may have influenced observed trends and are therefore interpreted as contextual rather than causal. Nonetheless, the comprehensive capture of all German hospital admissions minimizes selection bias. Our findings provide a realistic overview of ICU trends and outcomes, raising critical questions about intensive care utilization, capacity management, and ethical treatment thresholds.

## Conclusion

In conclusion, our study demonstrates substantial shifts in ICU utilization over the past decade in Germany. The decline in ICU admissions among patients older than 65 years of age suggests evolving selection practices, while increasing mortality among younger ICU patients warrants critical attention. ICU treatment in elderly and very elderly patients is associated with very poor outcomes including long-term cognitive and functional impairment, frequent rehospitalizations, and increased mortality [18, 21, 30]. In light of these findings and existing evidence on outcomes in elderly ICU patients, structured discussions about patient-centred outcomes and patient preferences may support alignment of treatment goals with individual values and may help to avoid burdensome interventions — sometimes resulting in a more cautious or watchful waiting approach [31]. With mandatory staffing regulations now in place, future trends in ICU admissions, outcomes, and IMC utilization will need close evaluation. Finally, reliance on ORTs as a "last resort" requires ongoing critical appraisal in light of poor outcomes across all age groups. Even among younger patients, careful assessment of prognosis, quality of life, and treatment burden remains essential before initiating invasive therapies—regardless of resource availability. While some degree of age-related selection may occur, individualised decision-making integrating clinical, functional, psychosocial, and patient preference factors remains key to providing optimal care.

## Supplementary Information

Supplementary Material 1

## Data Availability

The data used in this study are available from the GFSO upon reasonable request and subject to institutional data access agreements. Due to legal restrictions, the authors are not permitted to share the dataset directly.

## Abbreviations

CPAP: Continuous Positive Airway Pressure
DRG: Diagnosis-Related Groups
ECMO: Extracorporeal Membrane Oxygenation
GFSO: German Federal Statistical Office
ICD-10-GM: International Classification of Diseases 10th edition German Modification
ICU: Intensive Care Unit
IMC: Intermediate Care Unit
IQR: Interquartile Range
OPS: German Classification of Operations and Procedures
OR: Odds Ratio
ORT: Organ Replacement Therapy
RRT: Renal Replacement Therapy
VT: Mechanical ventilation
