# Supplementary material for: Decreasing admissions in older and increasing mortality in younger patients: a nationwide observational study of all German ICU cases 2011–2022
Source: Crit Care. 2026 Mar 6;30:159. doi: 10.1186/s13054-026-05924-y (PMC13063724; doi:10.1186/s13054-026-05924-y)
Supplement: Supplementary file 1 — Supplementary Material 1 [file 13054_2026_5924_MOESM1_ESM.docx]

**Supplemental Material**

to

**Decreasing admissions in older and increasing mortality in younger patients: A nationwide observational study of all German ICU cases from 2011–2022.**

*Critical Care*

Sarah-Yasmin Thomsen, MD^1^*, Nassim Kakavand, MD^2^*, Friedrich Alexander von Samson-Himmelstjerna, MD^1^, Ingo Eitel, MD^3^, Christian Jung, MD^4^, Roland Schmitt, MD^1^, Kevin Schulte, MD^1^, Benedikt Kolbrink, MD^1^

*1.* *Department of Nephrology and Hypertension, University Hospital Schleswig-Holstein Kiel, Christian-Albrechts University Kiel, Germany.*

*2. Department of Anaesthesiology and Operative Intensive Care Medicine, University Hospital Schleswig-Holstein Kiel, Christian-Albrechts University Kiel, Germany.*

*3. Department of Cardiology, Angiology and Intensive Care Medicine, University Hospital Schleswig-Holstein Lübeck, University of Lübeck, Lübeck, Germany.*

*4. Division of Cardiology, Pulmonary Diseases, Vascular Medicine, University Hospital Düsseldorf, Medical Faculty, University Düsseldorf, Germany*

* These authors contributed equally.

§ Corresponding author: Sarah-Yasmin Thomsen, MD

Department of Nephrology and Hypertension,

University Hospital Schleswig-Holstein Kiel,

Arnold-Heller-Str. 3

24105 Kiel, GERMANY

tel.no.: +49-431-500 64702

fax: +49-431-500 23044

mail: [sarah-yasmin.thomsen@uksh.de](mailto:sarah-yasmin.thomsen@uksh.de)

ORCID: 0009-0004-1365-4393

**Table of contents**

**Section**

[Table 1. ICD-10-GM codes used in the analyses 3](#_Toc220227196)

[Table 2. OPS codes used in the analyses 3](#_Toc220227197)

[Table 3. Reference years for the DRG statistics of the GFSO and list of R-packages used 4](#_Toc220227198)

[Table 4. Primary diagnoses of German ICU patients (2011–2022) 5](#_Toc220227199)

[Table 5. Top 20 primary causes of death in German ICUs stratified by age group (2011–2022) 7](#_Toc220227200)

[Table 6. ICU- and hospital admissions in Germany (2011–2022) 10](#_Toc220227201)

[Table 7. Mortality in German ICUs, hospitals and general population (2011–2022) 11](#_Toc220227202)

[Figure 1. ICU- and in-hospital deaths related to hospital admissions in Germany (2011–2022) 13](#_Toc220227203)

[Figure 2. ICU- and in-hospital deaths related to deaths in the German population (2011–2022) 14](#_Toc220227204)

[Figure 3. Sex- and age-specific incidence and mortality of ICU admissions in Germany (2011–2022) 15](#_Toc220227205)

[Table 8. Sex- and age-specific incidence of ICU admissions and deaths in Germany (2011–2022) 16](#_Toc220227206)

[Table 9. Incidence and mortality of organ replacement therapies in German ICUs (2011–2022) 18](#_Toc220227207)

[Figure 4. Incidence and mortality of organ replacement therapies in German ICUs (average across the observation period 2011–2022) 20](#_Toc220227208)

[Table 10. Use of single and combined organ replacement therapies in German ICUs (absolute numbers) (2011–2022) 21](#_Toc220227209)

[Table 11. Odds ratios for death with use of single and combined organ replacement therapies (2011–2022) 22](#_Toc220227210)

## Table 1. ICD-10-GM codes used in the analyses

| **corresponding ICD-10-GM codes** | **diagnosis** |
| --- | --- |
| A00–B99 | infectious and parasitic diseases |
| C00–D48 | neoplasms |
| D50–D90 | blood and immune disorders |
| E00–E90 | endocrine, nutritional and metabolic disorders |
| F00–F99 | mental and behavioral disorders |
| G00–G99 | nervous system disorders |
| H00–H95 | eye and ear disorders |
| I00–I99 | circulatory system disorders |
| J00–J99 | respiratory system disorders |
| K00–K93 | digestive system disorders |
| L00–L99 | skin and subcutaneous tissue disorders |
| M00–M99 | musculoskeletal and connective tissue disorders |
| N00–N99 | genitourinary disorders |
| V01–Y84, S00–T98 | external causes of morbidity and mortality |
| Z00–Z99, U00–U99 | other |

ICD-10-GM = International Classification of Diseases 10th Revision German Modification

## Table 2. OPS codes used in the analyses

| **procedure** | **duration** | **corresponding OPS codes** |
| --- | --- | --- |
| intensive care treatment   - *• Comprehensive intensive care treatment (adult)* - *• Comprehensive intensive care treatment in childhood (basic procedure)* - *• Advanced or highly complex intensive care treatment (basic procedure)* | - ≥24 h required | 8-980  8-98d  8-98f |
| mechanical ventilation (VT)   - Invasive mechanical ventilation: Invasive ventilation via endotracheal tube or tracheostomy;   includes prolonged ventilation and weaning   - Non-invasive ventilation (NIV): Non-invasive positive pressure ventilation via mask - Continuous Positive Airway Pressure (CPAP): Continuous positive airway pressure support   (newborns, children, adults) | - ≥24 h required | 8-70  8-71 |
| renal replacement therapy (RRT)   - Hemofiltration: Continuous or intermittent hemofiltration (arterio-venous / veno-venous) - Hemodialysis: Continuous or intermittent hemodialysis, including CVVHD - Hemodiafiltration: Continuous or intermittent hemodiafiltration (CAVHDF, CVVHDF) - Peritoneal dialysis: Intermittent or continuous peritoneal dialysis (IPD, CAPD, APD) | - any | 8-853 – 8-857 |
| extracorporeal membrane oxygenation (ECMO)   - Veno-venous (VV): Gas exchange without cardiac support - Veno-arterial (VA) / ECLS: Extracorporeal circulation with cardiac support   (ECLS = Extracorporeal Life Support)   - Other ECMO modalities: RA-PA ECMO (right atrium – pulmonary artery),   CO₂ removal (ECCO₂R), PECLA | - any | 8-852 |

OPS = “Operationen- und Prozedurenschlüssel”. German adaption of the international classification of procedures in medicine.

## Table 3. Reference years for the DRG statistics of the GFSO and list of R-packages used

| list of reference years of the DRG statistics of the GFSO used for analyses | - DRG-Statistik 2011, On-Site-Zugang; DOI: 10.21242/23141.2011.00.00.1.1.0 - DRG-Statistik 2012, On-Site-Zugang; DOI: 10.21242/23141.2012.00.00.1.1.0 - DRG-Statistik 2013, On-Site-Zugang; DOI: 10.21242/23141.2013.00.00.1.1.0 - DRG-Statistik 2014, On-Site-Zugang; DOI: 10.21242/23141.2014.00.00.1.1.0 - DRG-Statistik 2015, On-Site-Zugang; DOI: 10.21242/23141.2015.00.00.1.1.0 - DRG-Statistik 2016, On-Site-Zugang; DOI: 10.21242/23141.2016.00.00.1.1.0 - DRG-Statistik 2017, On-Site-Zugang; DOI: 10.21242/23141.2017.00.00.1.1.0 - DRG-Statistik 2018, On-Site-Zugang; DOI: 10.21242/23141.2018.00.00.1.1.0 - DRG-Statistik 2019, On-Site-Zugang; DOI: 10.21242/23141.2019.00.00.1.1.0 - DRG-Statistik 2020, On-Site-Zugang; DOI: 10.21242/23141.2020.00.00.1.1.0 - DRG-Statistik 2021, On-Site-Zugang; DOI: 10.21242/23141.2021.00.00.1.1.0 - DRG-Statistik 2022, On-Site-Zugang; DOI: 10.21242/23141.2022.00.00.1.1.0 |
| --- | --- |
| list of R-packages used for analyses | - Wickham H, Averick M, Bryan J, et al (2019) Welcome to the Tidyverse. J Open Source Softw 4:1686. https://doi.org/10.21105/joss.01686 - Sjoberg D D, Whiting K, Curry M, et al (2021) Reproducible Summary Tables with the gtsummary Package. R J 13:570. https://doi.org/10.32614/RJ-2021-053 - Hugh-Jones D (2017) huxtable: Easily Create and Style Tables for LaTeX, HTML and Other Formats. 5.6.0 - Ooms J (2017) writexl: Export Data Frames to Excel “xlsx” Format. 1.5.2 |

GFSO = “Federal Statistical Office of Germany”

## Table 4. Primary diagnoses of German ICU patients (2011–2022)

| **primary diagnosis** | **2011** | **2012** | **2013** | **2014** | **2015** | **2016** | **2017** | **2018** | **2019** | **2020** | **2021** | **2022** | **relative**  **change**  **[%]** | ***p*-value** |
| --- | --- | --- | --- | --- | --- | --- | --- | --- | --- | --- | --- | --- | --- | --- |
| all | 667,448 | 687,271 | 706,076 | 727,062 | 736,444 | 760,402 | 753,715 | 739,396 | 701,931 | 666,617 | 625,264 | 603,786 | -9.5% | 0.17 |
| blood and  immune  disorders | 3,095  (0.46%) | 2,837  (0.41%) | 2,899  (0.41%) | 3,076  (0.42%) | 3,028  (0.41%) | 2,852  (0.38%) | 2,840  (0.38%) | 2,706  (0.37%) | 2,551  (0.36%) | 2,423  (0.36%) | 2,020  (0.32%) | 2,137  (0.35%) | -31.0% | *<0.001* |
| infectious and  parasitic  diseases | 23,768  (3.56%) | 25,886  (3.77%) | 28,817  (4.08%) | 30,559  (4.20%) | 32,014  (4.35%) | 34,139  (4.49%) | 34,065  (4.52%) | 35,133  (4.75%) | 34,397  (4.90%) | 30,305  (4.55%) | 27,683  (4.43%) | 30,466  (5.05%) | +28.2% | 0.14 |
| circulatory  system  disorders | 264,640  (39.65%) | 271,596  (39.52%) | 273,073  (38.67%) | 280,164  (38.53%) | 279,927  (38.01%) | 285,725  (37.58%) | 280,227  (37.18%) | 268,527  (36.32%) | 253,189  (36.07%) | 233,205  (34.98%) | 208,927  (33.41%) | 207,196  (34.32%) | -21.7% | *0.005* |
| digestive  system  disorders | 72,353  (10.84%) | 73,247  (10.66%) | 72,941  (10.33%) | 74,449  (10.24%) | 73,254  (9.95%) | 75,851  (9.98%) | 74,329  (9.86%) | 72,303  (9.78%) | 69,009  (9.83%) | 64,782  (9.72%) | 57,975  (9.27%) | 58,562  (9.70%) | -19.1% | *0.003* |
| endocrine,  nutritional  and metabolic  disorders | 13,958  (2.09%) | 14,796  (2.15%) | 16,109  (2.28%) | 16,745  (2.30%) | 17,584  (2.39%) | 18,759  (2.47%) | 19,318  (2.56%) | 20,335  (2.75%) | 18,825  (2.68%) | 18,438  (2.77%) | 16,806  (2.69%) | 19,068  (3.16%) | +36.6% | *0.009* |
| external causes  of morbidity and  mortality | 74,971  (11.23%) | 75,426  (10.97%) | 77,052  (10.91%) | 81,025  (11.14%) | 81,456  (11.06%) | 83,156  (10.94%) | 82,058  (10.89%) | 81,757  (11.06%) | 75,809  (10.80%) | 71,323  (10.70%) | 62,135  (9.94%) | 65,192  (10.80%) | -13.0% | 0.06 |
| eye and ear  disorders | 713  (0.11%) | 721  (0.10%) | 782  (0.11%) | 750  (0.10%) | 866  (0.12%) | 1,037  (0.14%) | 894  (0.12%) | 896  (0.12%) | 736  (0.10%) | 596  (0.09%) | 482  (0.08%) | 459  (0.08%) | -35.7% | 0.12 |
| genitourinary  disorders | 17,835  (2.67%) | 18,849  (2.74%) | 19,765  (2.80%) | 20,169  (2.77%) | 20,380  (2.77%) | 21,369  (2.81%) | 20,822  (2.76%) | 20,749  (2.81%) | 19,855  (2.83%) | 19,546  (2.93%) | 17,295  (2.77%) | 17,960  (2.97%) | +0.7% | 0.64 |
| mental and  behavioral  disorders | 6,435  (0.96%) | 6,711  (0.98%) | 6,744  (0.96%) | 6,882  (0.95%) | 6,414  (0.87%) | 6,323  (0.83%) | 6,229  (0.83%) | 5,752  (0.78%) | 5,325  (0.76%) | 5,192  (0.78%) | 4,295  (0.69%) | 4,504  (0.75%) | -30.0% | *<0.001* |
| musculoskeletal  and connective  tissue disorders | 15,014  (2.25%) | 15,444  (2.25%) | 15,221  (2.16%) | 16,269  (2.24%) | 16,443  (2.23%) | 17,119  (2.25%) | 16,395  (2.18%) | 15,444  (2.09%) | 15,193  (2.16%) | 13,880  (2.08%) | 11,805  (1.89%) | 12,139  (2.01%) | -19.2% | *0.03* |
| neoplasms | 86,017  (12.89%) | 87,335  (12.71%) | 87,639  (12.41%) | 90,766  (12.48%) | 89,852  (12.20%) | 92,192  (12.12%) | 90,392  (11.99%) | 90,284  (12.21%) | 86,824  (12.37%) | 80,629  (12.10%) | 71,627  (11.46%) | 69,292  (11.48%) | -19.5% | *0.02* |
| nervous system  disorders | 17,775  (2.66%) | 18,454  (2.69%) | 19,223  (2.72%) | 20,107  (2.77%) | 20,307  (2.76%) | 22,091  (2.91%) | 22,005  (2.92%) | 21,112  (2.86%) | 19,141  (2.73%) | 17,546  (2.63%) | 15,966  (2.55%) | 16,069  (2.66%) | -9.6% | 0.29 |
| other | 15,413  (2.31%) | 18,236  (2.65%) | 19,665  (2.79%) | 21,201  (2.92%) | 20,866  (2.83%) | 21,769  (2.86%) | 21,638  (2.87%) | 20,946  (2.83%) | 19,909  (2.84%) | 18,336  (2.75%) | 17,066  (2.73%) | 16,809  (2.78%) | +9.0% | 0.84 |
| respiratory  system  disorders | 53,356  (7.99%) | 55,534  (8.08%) | 63,785  (9.03%) | 62,382  (8.58%) | 71,413  (9.70%) | 75,351  (9.91%) | 79,751  (10.58%) | 80,769  (10.92%) | 78,489  (11.18%) | 88,021  (13.20%) | 109,051  (17.44%) | 81,668  (13.53%) | +53.1% | *<0.001* |
| skin and  subcutaneous  tissue disorders | 2,105  (0.32%) | 2,199  (0.32%) | 2,361  (0.33%) | 2,518  (0.35%) | 2,640  (0.36%) | 2,669  (0.35%) | 2,752  (0.37%) | 2,683  (0.36%) | 2,679  (0.38%) | 2,395  (0.36%) | 2,131  (0.34%) | 2,265  (0.38%) | +7.6% | 0.69 |

## Table 5. Top 20 primary causes of death in German ICUs stratified by age group (2011–2022)

|  | **ICD-10 code** | **corresponding diagnosis** | **total numbers (%)** |
| --- | --- | --- | --- |
| **all ICU deaths** | | | 1,307,677 (100%) |
| 1. | I50 | heart failure | 87,143 (6.7%) |
| 2. | I21 | acute myocardial infarction | 81,284 (6.2%) |
| 3. | A41 | sepsis | 75,360 (5.8%) |
| 4. | I63 | cerebral infarction | 49,387 (3.8%) |
| 5. | J18 | pneumonia, unspecified organism | 42,001 (3.2%) |
| 6. | J44 | other chronic obstructive pulmonary disease (COPD) | 41,163 (3.1%) |
| 7. | S72 | fracture of femur | 33,756 (2.6%) |
| 8. | J12 | viral pneumonia, not elsewhere classified | 32,680 (2.5%) |
| 9. | I61 | intracerebral hemorrhage | 31,413 (2.4%) |
| 10. | S06 | intracranial injury | 31,349 (2.4%) |
| 11. | N17 | acute renal failure | 26,449 (2.0%) |
| 12. | J15 | bacterial pneumonia, not elsewhere classified | 22,252 (1.7%) |
| 13. | I70 | atherosclerosis | 20,267 (1.5%) |
| 14. | C34 | malignant neoplasm of bronchus and lung | 20,108 (1.5%) |
| 15. | K56 | paralytic ileus and intestinal obstruction | 19,898 (1.5%) |
| 16. | J96 | respiratory failure, not elsewhere classified | 19,330 (1.5%) |
| 17. | K70 | alcoholic liver disease | 17,179 (1.3%) |
| 18. | C18 | malignant neoplasm of colon | 15,826 (1.2%) |
| 19. | I46 | cardiac arrest | 15,584 (1.2%) |
| 20. | I35 | nonrheumatic aortic valve disorders | 15,302 (1.2%) |
|  | **ICD-10 code** | **corresponding diagnosis** | **total numbers (%)** |
| **deaths <65 years** | | | 280,497 (100%) |
| 1. | A41 | sepsis | 16,956 (6.0%) |
| 2. | I21 | acute myocardial infarction | 16,049 (5.7%) |
| 3. | K70 | alcoholic liver disease | 12,895 (4.6%) |
| 4. | I50 | heart failure | 8,649 (3.1%) |
| 5. | I61 | intracerebral hemorrhage | 7,790 (2.8%) |
| 6. | I63 | cerebral infarction | 7,430 (2.6%) |
| 7. | C34 | malignant neoplasm of bronchus and lung | 7,100 (2.5%) |
| 8. | J44 | other chronic obstructive pulmonary disease (COPD) | 6,936 (2.5%) |
| 9. | I60 | subarachnoid hemorrhage | 6,805 (2.4%) |
| 10. | J12 | viral pneumonia, not elsewhere classified | 6,802 (2.4%) |
| 11. | S06 | intracranial injury | 6,419 (2.3%) |
| 12. | J18 | pneumonia, unspecified organism | 6,091 (2.2%) |
| 13. | J80 | acute respiratory distress syndrome (ARDS) | 5,245 (1.9%) |
| 14. | I46 | cardiac arrest | 4,799 (1.7%) |
| 15. | J96 | respiratory failure, not elsewhere classified | 4,116 (1.5%) |
| 16. | J15 | bacterial pneumonia, not elsewhere classified | 4,115 (1.5%) |
| 17. | I49 | other cardiac arrhythmias | 3,349 (1.2%) |
| 18. | R57 | shock, not elsewhere classified | 3,339 (1.2%) |
| 19. | N17 | acute renal failure | 3,305 (1.2%) |
| 20. | C92 | myeloid leukemia | 3,266 (1.2%) |
|  | **ICD-10 code** | **corresponding diagnosis** | **total numbers (%)** |
| **deaths 65–79 years** | | | 556,012 (100%) |
| 1. | I50 | heart failure | 36,168 (6.5%) |
| 2. | I21 | acute myocardial infarction | 36,036 (6.5%) |
| 3. | A41 | sepsis | 33,824 (6.1%) |
| 4. | J44 | other chronic obstructive pulmonary disease (COPD) | 22,930 (4.1%) |
| 5. | I63 | cerebral infarction | 20,339 (3.7%) |
| 6. | J18 | pneumonia, unspecified organism | 17,828 (3.2%) |
| 7. | J12 | viral pneumonia, not elsewhere classified | 15,063 (2.7%) |
| 8. | I61 | intracerebral hemorrhage | 14,107 (2.5%) |
| 9. | N17 | acute renal failure | 11,631 (2.1%) |
| 10. | C34 | malignant neoplasm of bronchus and lung | 10,839 (1.9%) |
| 11. | J15 | bacterial pneumonia, not elsewhere classified | 10,282 (1.8%) |
| 12. | S06 | intracranial injury | 9,979 (1.8%) |
| 13. | I70 | atherosclerosis | 9,671 (1.7%) |
| 14. | J96 | respiratory failure, not elsewhere classified | 9,417 (1.7%) |
| 15. | S72 | fracture of femur | 7,179 (1.3%) |
| 16. | K56 | paralytic ileus and intestinal obstruction | 6,913 (1.2%) |
| 17. | I35 | nonrheumatic aortic valve disorders | 6,885 (1.2%) |
| 18. | I71 | aortic aneurysm and dissection | 6,827 (1.2%) |
| 19. | I46 | cardiac arrest | 6,549 (1.2%) |
| 20. | C18 | malignant neoplasm of colon | 6,281 (1.1%) |
|  | **ICD-10 code** | **corresponding diagnosis** | **total numbers (%)** |
| **deaths ≥80 years** | | | 471,168 (100%) |
| 1. | I50 | heart failure | 42,326 (9.0%) |
| 2. | I21 | acute myocardial infarction | 29,199 (6.2%) |
| 3. | S72 | fracture of femur | 25,242 (5.4%) |
| 4. | A41 | sepsis | 24,580 (5.2%) |
| 5. | I63 | cerebral infarction | 21,618 (4.6%) |
| 6. | J18 | pneumonia, unspecified organism | 18,082 (3.8%) |
| 7. | S06 | Intracranial injury | 14,951 (3.2%) |
| 8. | N17 | acute renal failure | 11,513 (2.4%) |
| 9. | J44 | other chronic obstructive pulmonary disease (COPD) | 11,297 (2.4%) |
| 10. | J12 | viral pneumonia, not elsewhere classified | 10,815 (2.3%) |
| 11. | K56 | paralytic ileus and intestinal obstruction | 10,704 (2.3%) |
| 12. | I61 | intracerebral hemorrhage | 9,516 (2.0%) |
| 13. | I70 | atherosclerosis | 7,995 (1.7%) |
| 14. | C18 | malignant neoplasm of colon | 7,885 (1.7%) |
| 15. | J15 | bacterial pneumonia, not elsewhere classified | 7,855 (1.7%) |
| 16. | I35 | nonrheumatic aortic valve disorders | 7,105 (1.5%) |
| 17. | K55 | vascular disorders of intestine | 6,937 (1.5%) |
| 18. | J69 | pneumonitis due to solids and liquids (aspiration) | 6,120 (1.3%) |
| 19. | J96 | respiratory failure, not elsewhere classified | 5,797 (1.2%) |
| 20. | K57 | diverticular disease of intestine | 5,183 (1.1%) |

ICD-10-GM = International Classification of Diseases 10th Revision German Modification

## Table 6. ICU- and hospital admissions in Germany (2011–2022)

|  | | **2011** | **2012** | **2013** | **2014** | **2015** | **2016** | **2017** | **2018** | **2019** | **2020** | **2021** | **2022** | **relative**  **change**  **[%]** | ***p*-value** |
| --- | --- | --- | --- | --- | --- | --- | --- | --- | --- | --- | --- | --- | --- | --- | --- |
| ICU  admissions  [total  numbers  (%)] | general  population | 667,448  (100) | 687,271  (100) | 720,761  (100) | 727,062  (100) | 736,444  (100) | 760,402  (100) | 753,715  (100) | 739,396  (100) | 701,931  (100) | 666,617  (100) | 625,264  (100) | 603,786  (100) | -9.5 | 0.17 |
|  | < 18  years | 15,790  (2.4) | 16,798  (2.4) | 17,265  (2.5) | 17,808  (2.5) | 18,910  (2.6) | 19,732  (2.6) | 19,886  (2.6) | 19,412  (2.6) | 19,327  (2.8) | 18,122  (2.7) | 17,483  (2.8) | 15,125  (2.5) | -4.2 | 0.72 |
|  | < 65  years | 248,989  (37.3) | 255,401  (37.2) | 262,974  (37.2) | 269,812  (37.1) | 272,886  (37.1) | 281,869  (37.1) | 275,488  (36.6) | 271,737  (36.8) | 256,896  (36.6) | 247,272  (37.1) | 240,123  (38.4) | 224,316  (37.2) | -9.9 | 0.15 |
|  | 65-79  years | 277,817  (41.6) | 285,038  (41.5) | 290,980  (41.2) | 299,443  (41.2) | 300,288  (40.8) | 305,565  (40.2) | 300,213  (39.8) | 291,411  (39.4) | 272,921  (38.9) | 252,950  (37.9) | 233,817  (37.4) | 227,196  (37.6) | -18.2 | *0.01* |
|  | ≥ 80  years | 140,642  (21.1) | 146,832  (21.4) | 152,122  (21.5) | 157,807  (21.7) | 163,270  (22.2) | 172,968  (22.7) | 178,014  (23.6) | 176,248  (23.8) | 172,114  (24.5) | 166,395  (25.0) | 151,324  (24.2) | 152,274  (25.2) | +8.3 | 0.21 |
| ICU  admissions  /  100.000  inhabitants | general  population | 830.9 | 853.5 | 874.2 | 895.4 | 896.2 | 921.5 | 910.4 | 890.6 | 844.0 | 801.7 | 751.2 | 726.4 | -12.6 | *0.05* |
|  | < 18  years | 119.6 | 128.1 | 132.0 | 136.0 | 143.1 | 147.3 | 147.3 | 143.1 | 141.7 | 132.2 | 126.7 | 110.2 | -7.9 | 0.77 |
|  | < 65  years | 391.0 | 399.9 | 407.8 | 421.5 | 423.1 | 434.0 | 423.5 | 417.4 | 394.6 | 380.5 | 370.3 | 349.7 | -10.6 | 0.07 |
|  | 65-79  years | 2,247.0 | 2,305.8 | 2,339.5 | 2,392.6 | 2,391.3 | 2,431.0 | 2,389.6 | 2,326.3 | 2,191.8 | 2,044.5 | 1,896.3 | 1,839.2 | -18.1 | *0.008* |
|  | ≥ 80  years | 3,327.9 | 3,416.7 | 3,500.2 | 3,542.0 | 3,521.2 | 3,577.0 | 3,527.6 | 3,344.4 | 3,109.5 | 2,864.5 | 2,512.0 | 2,527.4 | -24.0 | *0.003* |
| hospital  admissions  [total  numbers  (%)] | general  population | 18,797,884  (100) | 19,082,299  (100) | 19,249,305  (100) | 19,632,754  (100) | 19,758,260  (100) | 20,063,687  (100) | 19,952,725  (100) | 19,801,851  (100) | 19,855,783  (100) | 17,265,142  (100) | 17,157,549  (100) | 17,205,578  (100) | -8.5 | 0.08 |
|  | < 65  years | 10,745,732  (57.2) | 10,866,876  (56.9) | 10,938,703  (56.8) | 11,158,779  (56.8) | 11,149,091  (56.4) | 11,349,439  (56.6) | 11,151,551  (55.9) | 10,997,065  (55.5) | 10,933,304  (55.1) | 9,471,468  (54.9) | 9,455,208  (55.1) | 9,277,936  (53.9) | -13.7 | *0.01* |
|  | 65-79  years | 5,244,840  (27.9) | 5,307,813  (27.8) | 5,331,974  (27.7) | 5,413,101  (27.6) | 5,405,104  (27.4) | 5,396,521  (26.9) | 5,342,054  (26.8) | 5,245,456  (26.5) | 5,195,927  (26.2) | 4,426,026  (25.6) | 4,280,908  (25.0) | 4,342,419  (25.2) | -17.2 | *0.004* |
|  | ≥ 80  years | 2,807,312  (14.9) | 2,907,610  (15.2) | 2,978,628  (15.5) | 3,060,874  (15.6) | 3,204,065  (16.2) | 3,317,727  (16.5) | 3,459,120  (17.3) | 3,559,330  (18.0) | 3,726,552  (18.8) | 3,367,648  (19.5) | 3,421,433  (19.9) | 3,585,223  (20.8) | +27.7 | *<*  *0.001* |
| hospital  admissions  /  100.000  inhabitants | general  population | 23,401.4 | 23,697.7 | 23,833.0 | 24,179.0 | 24,043.9 | 24,313.2 | 24,099.7 | 23,852.1 | 23,874.7 | 20,762.6 | 20,612.9 | 20,700.1 | -11.5 | *0.01* |
|  | < 65  years | 16,873.2 | 17,016.3 | 16,961.3 | 17,432.4 | 17,287.5 | 17,475.8 | 17,143.8 | 16,890.2 | 16,793.1 | 14,576.0 | 14,581.9 | 14,462.6 | -14.3 | *0.005* |
|  | 65-79  years | 42,420.8 | 42,937.0 | 42,868.6 | 43,251.4 | 43,042.2 | 42,933.8 | 42,520.7 | 41,874.1 | 41,727.7 | 35,773.4 | 34,719.4 | 35,152.3 | -17.1 | *0.002* |
|  | ≥ 80  years | 66,427.8 | 67,658.9 | 68,536.4 | 68,701.3 | 69,101.5 | 68,610.9 | 68,547.5 | 67,540.8 | 67,325.6 | 57,975.1 | 56,796.3 | 59,506.9 | -10.4 | *0.010* |
| ICU  admissions  /  hospital  admissions  [%] | general  population | 3.6 | 3.6 | 3.7 | 3.7 | 3.7 | 3.8 | 3.8 | 3.7 | 3.5 | 3.9 | 3.6 | 3.5 | -2.8 | 0.88 |
|  | < 65  years | 2.3 | 2.4 | 2.4 | 2.4 | 2.5 | 2.5 | 2.5 | 2.5 | 2.4 | 2.6 | 2.5 | 2.4 | +4.3 | *0.04* |
|  | 65-79  years | 5.3 | 5.4 | 5.5 | 5.5 | 5.6 | 5.7 | 5.6 | 5.6 | 5.3 | 5.7 | 5.5 | 5.2 | -1.3 | 0.92 |
|  | ≥ 80  years | 5.0 | 5.1 | 5.1 | 5.2 | 5.1 | 5.2 | 5.2 | 5.0 | 4.6 | 4.9 | 4.4 | 4.3 | -15.2 | *0.006* |

ICU = intensive care unit

## Table 7. Mortality in German ICUs, hospitals and general population (2011–2022)

| **mortality in German ICUs** | | | | | | | | | | | | | | | | | | | | | | | | | | | | |
| --- | --- | --- | --- | --- | --- | --- | --- | --- | --- | --- | --- | --- | --- | --- | --- | --- | --- | --- | --- | --- | --- | --- | --- | --- | --- | --- | --- | --- |
|  | | | **2011** | **2012** | **2013** | | **2014** | | **2015** | | **2016** | | **2017** | | **2018** | | **2019** | | **2020** | | **2021** | | **2022** | | **relative**  **change**  **[%]** | | ***p*-value** | |
| ICU deaths  [total numbers  (%)] | general  population | | 96,146  (100) | 97,924  (100) | 103,322  (100) | | 103,030  (100) | | 108,866  (100) | | 111,062  (100) | | 113,392  (100) | | 113,800  (100) | | 110,902  (100) | | 113,014  (100) | | 121,979  (100) | | 114,240  (100) | | +18.8 | | *<0.001* | |
|  | < 65 years | | 20,956  (21.8) | 20,805  (21.2) | 22,035  (21.3) | | 22,039  (21.4) | | 22,962  (21.1) | | 23,798  (21.4) | | 23,353  (20.6) | | 24,350  (21.4) | | 23,307  (21.0) | | 23,998  (21.2) | | 27,751  (22.8) | | 25,143  (22.0) | | +20 | | *<0.001* | |
|  | 65-79 years | | 42,770  (44.5) | 43,137  (44.1) | 45,372  (43.9) | | 45,332  (44.0) | | 47,435  (43.6) | | 47,681  (42.9) | | 48,159  (42.5) | | 47,776  (42.0) | | 45,742  (41.2) | | 46,076  (40.8) | | 50,638  (41.5) | | 45,894  (40.2) | | +7.3 | | *0.03* | |
|  | ≥ 80 years | | 32,420  (33.7) | 33,982  (34.7) | 35,915  (34.8) | | 35,659  (34.6) | | 38,469  (35.3) | | 39,583  (35.6) | | 41,880  (36.9) | | 41,674  (36.6) | | 41,853  (37.7) | | 42,940  (38.0) | | 43,590  (35.7) | | 43,203  (37.8) | | +33.3 | | *<0.001* | |
| ICU deaths  /  100.000  inhabitants | general  population | | 119.7 | 121.6 | 127.9 | | 126.9 | | 132.5 | | 134.6 | | 137.0 | | 137.1 | | 133.3 | | 135.9 | | 146.5 | | 137.4 | | +14.8 | | *<0.001* | |
|  | < 65 years | | 32.9 | 32.6 | 34.2 | | 34.4 | | 35.6 | | 36.6 | | 35.9 | | 37.4 | | 35.8 | | 36.9 | | 42.8 | | 39.2 | | +19.2 | | *<0.001* | |
|  | 65-79 years | | 345.9 | 349.0 | 364.8 | | 362.2 | | 377.7 | | 379.3 | | 383.3 | | 381.4 | | 367.3 | | 372.4 | | 410.7 | | 371.5 | | +7.4 | | *0.01* | |
|  | ≥ 80 years | | 767.1 | 790.7 | 826.4 | | 800.4 | | 829.7 | | 818.6 | | 829.9 | | 790.8 | | 756.1 | | 739.2 | | 723.6 | | 717.1 | | -6.5 | | *0.03* | |
| ICU mortality  [% of ICU  admissions] | general  population | | 14.4 | 14.2 | 14.6 | | 14.2 | | 14.8 | | 14.6 | | 15.0 | | 15.4 | | 15.8 | | 17.0 | | 19.5 | | 18.9 | | +31.3 | | *<0.001* | |
|  | < 65 years | | 8.4 | 8.1 | 8.4 | | 8.2 | | 8.4 | | 8.4 | | 8.5 | | 9.0 | | 9.1 | | 9.7 | | 11.6 | | 11.2 | | +33.3 | | *<0.001* | |
|  | 65-79 years | | 15.4 | 15.1 | 15.6 | | 15.1 | | 15.8 | | 15.6 | | 16.0 | | 16.4 | | 16.8 | | 18.2 | | 21.7 | | 20.2 | | +31.2 | | *<0.001* | |
|  | ≥ 80 years | | 23.1 | 23.1 | 23.6 | | 22.6 | | 23.6 | | 22.9 | | 23.5 | | 23.6 | | 24.3 | | 25.8 | | 28.8 | | 28.4 | | +22.9 | | *0.002* | |
| ICU deaths  /  100 hospital  admissions | all hospital  admissions | | 0.51 | 0.51 | 0.54 | | 0.52 | | 0.55 | | 0.55 | | 0.57 | | 0.57 | | 0.56 | | 0.65 | | 0.71 | | 0.66 | | +29.4 | | *<0.001* | |
|  | < 65 years | | 0.20 | 0.19 | 0.20 | | 0.20 | | 0.21 | | 0.21 | | 0.21 | | 0.22 | | 0.21 | | 0.25 | | 0.29 | | 0.27 | | +35.0 | | *<0.001* | |
|  | 65-79 years | | 0.82 | 0.81 | 0.85 | | 0.84 | | 0.88 | | 0.88 | | 0.90 | | 0.91 | | 0.88 | | 1.04 | | 1.18 | | 1.06 | | +29.3 | | *<0.001* | |
|  | ≥ 80 years | | 1.15 | 1.17 | 1.21 | | 1.16 | | 1.20 | | 1.19 | | 1.21 | | 1.17 | | 1.12 | | 1.28 | | 1.27 | | 1.21 | | +5.2 | | 0.11 | |
| ICU deaths  /  In-hospital  deaths  [%] | general  population | | 23.9 | 24.2 | 24.8 | | 25.5 | | 25.5 | | 26.5 | | 26.5 | | 26.0 | | 26.0 | | 26.6 | | 27.3 | | 25.0 | | +4.3 | | *0.01* | |
|  | < 65 years | | 29.5 | 29.9 | 31.0 | | 31.9 | | 32.4 | | 33.7 | | 33.8 | | 33.5 | | 34.1 | | 35.6 | | 38.8 | | 35.9 | | +21.8 | | *<0.001* | |
|  | 65-79 years | | 28.4 | 28.9 | 29.6 | | 30.5 | | 30.8 | | 32.0 | | 32.4 | | 32.1 | | 32.0 | | 33.4 | | 35.1 | | 32.0 | | +12.6 | | *<0.001* | |
|  | ≥ 80 years | | 18.0 | 18.3 | 18.6 | | 19.2 | | 19.0 | | 19.8 | | 20.0 | | 19.3 | | 19.4 | | 19.6 | | 18.8 | | 17.7 | | -1.7 | | 0.56 | |
| ICU deaths  /  100 deaths  in Germany | general  population | | 11.3 | 11.3 | 11.6 | | 11.9 | | 11.8 | | 12.2 | | 12.2 | | 11.9 | | 11.8 | | 11.5 | | 11.9 | | 10.7 | | -5.1 | | 0.85 | |
|  | < 65 years | | 15.1 | 15.2 | 15.8 | | 16.1 | | 16.3 | | 17.0 | | 17.1 | | 17.4 | | 17.2 | | 17.4 | | 19.2 | | 17.6 | | +16.6 | | *<0.001* | |
|  | 65-79 years | | 15.7 | 15.7 | 16.1 | | 16.6 | | 16.7 | | 17.1 | | 17.4 | | 17.2 | | 17.0 | | 17.1 | | 18.3 | | 16.4 | | +4.5 | | *0.010* | |
|  | ≥ 80 years | | 7.4 | 7.4 | 7.6 | | 7.8 | | 7.7 | | 8.0 | | 8.1 | | 7.8 | | 7.8 | | 7.4 | | 7.2 | | 6.7 | | -9.5 | | 0.32 | |
| **mortality in German hospitals and in the German general population** | | | | | | | | | | | | | | | | | | | | | | | | | | | | |
|  | | | **2011** | **2012** | | **2013** | | **2014** | | **2015** | | **2016** | | **2017** | | **2018** | | **2019** | | **2020** | | **2021** | | **2022** | | **relative**  **change**  **[%]** | | ***p*-value** |
| in-hospital  deaths  [total numbers  (%)] | | general  population | 401,865  (100) | 404,842  (100) | | 417,290  (100) | | 403,787  (100) | | 427,201  (100) | | 419,359  (100) | | 427,917  (100) | | 437,220  (100) | | 427,199  (100) | | 424,635  (100) | | 447,473  (100) | | 457,743  (100) | | +13.9 | | *<0.001* |
|  |  | < 65 years | 71,159  (17.7) | 69,482  (17.2) | | 70,989  (17.0) | | 69,109  (17.1) | | 70,891  (16.6) | | 70,561  (16.8) | | 69,190  (16.2) | | 72,638  (16.6) | | 68,324  (16.0) | | 67,393  (15.9) | | 71,540  (16.0) | | 70,096  (15.3) | | -1.5 | | 0.63 |
|  |  | 65-79 years | 150,391  (37.4) | 149,253  (36.9) | | 153,357  (36.8) | | 148,526  (36.8) | | 153,790  (36.0) | | 149,235  (35.6) | | 148,760  (34.8) | | 149,027  (34.1) | | 142,872  (33.4) | | 137,949  (32.5) | | 144,349  (32.3) | | 143,221  (31.3) | | -4.8 | | *0.005* |
|  |  | ≥ 80 years | 180,315  (44.9) | 186,107  (46.0) | | 192,944  (46.2) | | 186,152  (46.1) | | 202,520  (47.4) | | 199,563  (47.6) | | 209,967  (49.1) | | 215,555  (49.3) | | 216,003  (50.6) | | 219,293  (51.6) | | 231,584  (51.8) | | 244,426  (53.4) | | +35.6 | | *<0.001* |
| in-hospital  deaths  /  100.000  inhabitants | | general  population | 500.3 | 502.8 | | 516.7 | | 497.3 | | 519.9 | | 508.2 | | 516.9 | | 526.6 | | 513.7 | | 510.7 | | 537.6 | | 550.7 | | +10.1 | | *0.003* |
|  |  | < 65 years | 111.7 | 108.8 | | 110.1 | | 108.0 | | 109.9 | | 108.6 | | 106.4 | | 111.6 | | 104.9 | | 103.7 | | 110.3 | | 109.3 | | -2.2 | | 0.26 |
|  |  | 65-79 years | 1,216.4 | 1,207.4 | | 1,233.0 | | 1,186.7 | | 1,224.7 | | 1,187.3 | | 1,184.1 | | 1,189.7 | | 1,147.4 | | 1,115.0 | | 1,170.7 | | 1,159.4 | | -4.7 | | *0.003* |
|  |  | ≥ 80 years | 4,266.7 | 4,330.6 | | 4,439.5 | | 4,178.2 | | 4,367.7 | | 4,127.0 | | 4,160.8 | | 4,090.3 | | 3,902.4 | | 3,775.2 | | 3,844.3 | | 4,056.9 | | -4.9 | | *0.001* |
| in-hospital  deaths  /  100 hospital  admissions | | all hospital  admissions | 2.1 | 2.1 | | 2.2 | | 2.1 | | 2.2 | | 2.1 | | 2.1 | | 2.2 | | 2.2 | | 2.5 | | 2.6 | | 2.7 | | +24.3 | | *0.003* |
|  |  | < 65 years | 0.7 | 0.6 | | 0.6 | | 0.6 | | 0.6 | | 0.6 | | 0.6 | | 0.7 | | 0.6 | | 0.7 | | 0.8 | | 0.8 | | +15.2 | | *0.02* |
|  |  | 65-79 years | 2.9 | 2.8 | | 2.9 | | 2.7 | | 2.8 | | 2.8 | | 2.8 | | 2.8 | | 2.7 | | 3.1 | | 3.4 | | 3.3 | | +15.0 | | *0.02* |
|  |  | ≥ 80 years | 6.4 | 6.4 | | 6.5 | | 6.1 | | 6.3 | | 6.0 | | 6.1 | | 6.1 | | 5.8 | | 6.5 | | 6.8 | | 6.8 | | +6.2 | | 0.46 |
| German  deaths  [total numbers  (%)] | | general  population | 852,328  (100) | 869,582  (100) | | 893,825  (100) | | 868,356  (100) | | 925,200  (100) | | 910,899  (100) | | 932,263  (100) | | 954,874  (100) | | 939,520  (100) | | 985,572  (100) | | 1,023,687  (100) | | 1,066,341  (100) | | +25.1 | | *<0.001* |
|  |  | < 65 years | 138,419  (16.2) | 137,074  (15.8) | | 139,516  (15.6) | | 136,560  (15.7) | | 140,835  (15.2) | | 139,703  (15.3) | | 136,905  (14.7) | | 139,582  (14.6) | | 135,567  (14.4) | | 137,592  (14.0) | | 144,340  (14.1) | | 142,747  (13.4) | | +3.1 | | 0.17 |
|  |  | 65-79 years | 273,096  (32.0) | 274,180  (31,5) | | 281,454  (31.5) | | 273,690  (31.5) | | 284,454  (30.7) | | 278,036  (30.5) | | 277,550  (29.8) | | 278,397  (29.2) | | 268,459  (28.6) | | 269,481  (27.3) | | 276,839  (27.0) | | 280,228  (26.3) | | +2.6 | | 0.83 |
|  |  | ≥ 80 years | 440,813  (51.7) | 458,328  (52.7) | | 472,855  (52.9) | | 458,106  (52.8) | | 499,911  (54.0) | | 493,160  (54.1) | | 517,808  (55.5) | | 536,895  (56.2) | | 535,494  (57.0) | | 578,499  (58.7) | | 602,508  (58.9) | | 643,366  (60.3) | | +46.0 | | *<0.001* |
| in-hospital  deaths  /  100 deaths  in Germany | | general  population | 47.2 | 46.6 | | 46.7 | | 46.5 | | 46.2 | | 46.0 | | 45.9 | | 45.8 | | 45.5 | | 43.1 | | 43.7 | | 42.9 | | -9.0 | | *<0.001* |
|  |  | < 65 years | 51.4 | 50.7 | | 50.9 | | 50.6 | | 50.3 | | 50.5 | | 50.5 | | 52.0 | | 50.4 | | 49.0 | | 49.6 | | 49.1 | | -4.5 | | *0.02* |
|  |  | 65-79 years | 55.1 | 54.4 | | 54.5 | | 54.3 | | 54.1 | | 53.7 | | 53.6 | | 53.5 | | 53.2 | | 51.2 | | 52.1 | | 51.1 | | -7.3 | | *<0.001* |
|  |  | ≥ 80 years | 40.9 | 40.6 | | 40.8 | | 40.6 | | 40.5 | | 40.5 | | 40.5 | | 40.1 | | 40.3 | | 37.9 | | 38.4 | | 38.0 | | -7.1 | | *<0.001* |

ICU = intensive care unit

## Figure 1. ICU- and in-hospital deaths related to hospital admissions in Germany (2011–2022)


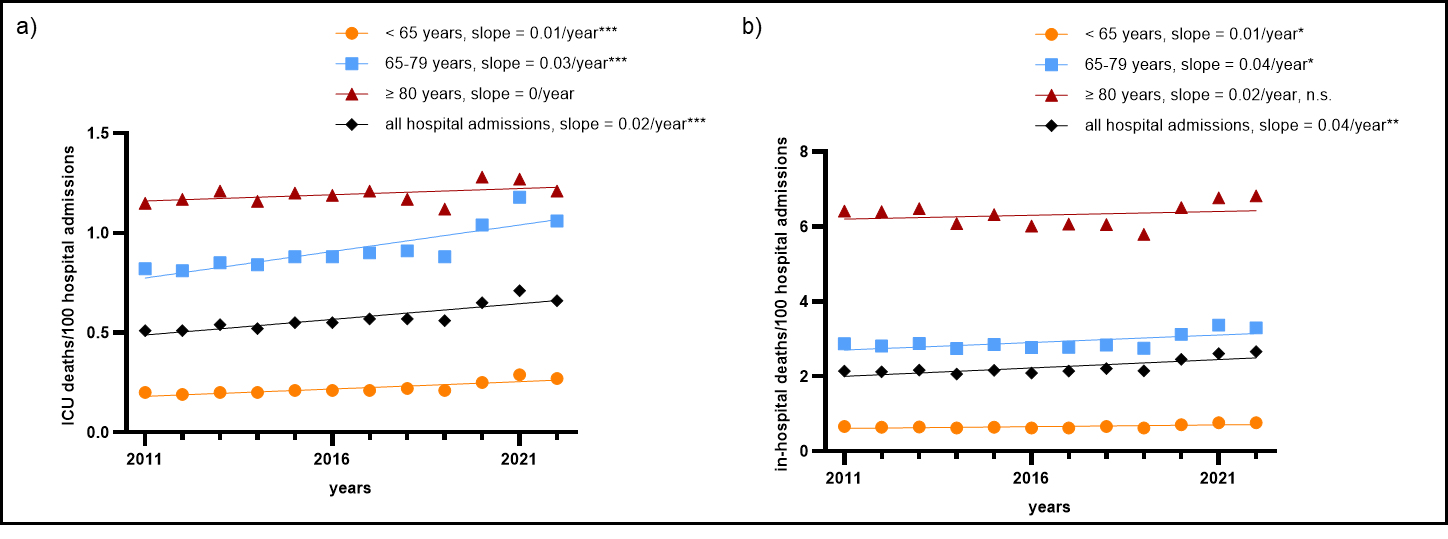


The panel a) shows the number of ICU deaths per 100 hospital admissions, overall and stratified by age group. The panel b) displays the number of in-hospital deaths per 100 hospital admissions, overall and stratified by age group. Significance levels: * = p < 0.05; ** = p < 0.01; *** = p < 0.001; n.s. = not significant (p ≥ 0.05).

## Figure 2. ICU- and in-hospital deaths related to deaths in the German population (2011–2022)


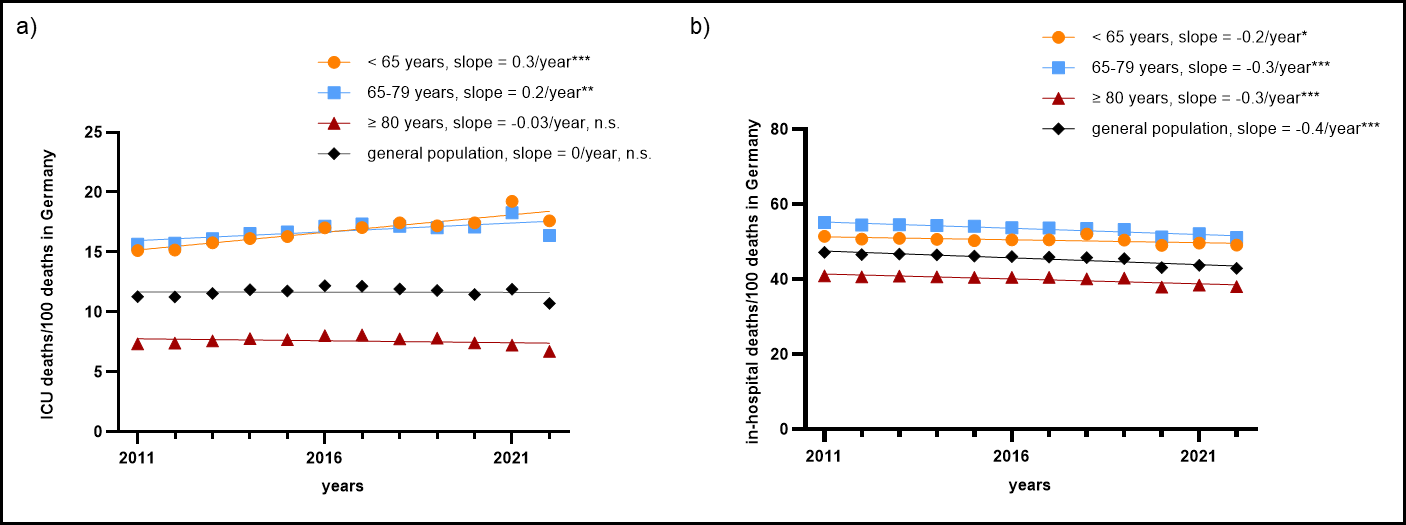


*The panel a) presents changes over time in the number of ICU deaths among all deaths and stratified by age group related to 100 deaths in the German general population. The panel b) shows the number of in-hospital deaths over the study period, stratified by age group and related to 100 deaths in the German general population. Significance levels: * = p < 0.05; ** = p < 0.01; *** = p < 0.001; n.s. = not significant (p ≥ 0.05).*

## Figure 3. Sex- and age-specific incidence and mortality of ICU admissions in Germany (2011–2022)


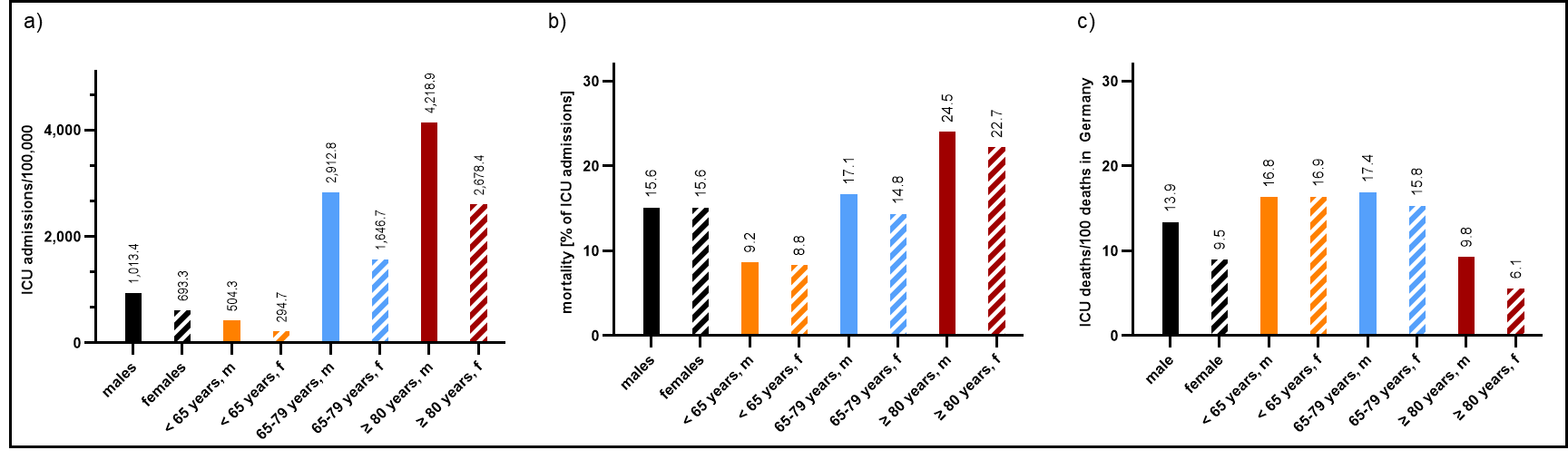


*The panel a) shows incidence of ICU admissions stratified by sex and age averaged across the observation period from 2011 to 2022. The panels b) and c) display mortality in different sex and age groups, related to ICU admissions and to all deaths in Germany.*

## Table 8. Sex- and age-specific incidence of ICU admissions and deaths in Germany (2011–2022)

| **sex and age-specific incidence of ICU admissions in Germany** | | | | | | | | | | | | | | | |
| --- | --- | --- | --- | --- | --- | --- | --- | --- | --- | --- | --- | --- | --- | --- | --- |
|  | | **2011** | **2012** | **2013** | **2014** | **2015** | **2016** | **2017** | **2018** | **2019** | **2020** | **2021** | **2022** | **relative**  **change**  **[%]** | ***p*-value** |
| male ICU  admissions  [total numbers  (%)] | general  population | 386,235  (100) | 398,534  (100) | 410,414  (100) | 422,803  (100) | 429,583  (100) | 443,066  (100) | 439,874  (100) | 433,522  (100) | 412,973  (100) | 395,885  (100) | 374,854  (100) | 360,221  (100) | -6.7 | 0.33 |
|  | < 65 years | 159,444  (41.3) | 163,120  (40.9) | 167,714  (40.9) | 172,207  (40.7) | 173,853  (40.5) | 179,446  (40.5) | 174,993  (39.8) | 172,533  (39.8) | 163,134  (39.5) | 158,558  (40.1) | 154,908  (41.3) | 143,471  (39.8) | -10.0 | 0.14 |
|  | 65-79 years | 166,657  (43.1) | 171,198  (43.0) | 174,477  (42.5) | 179,547  (42.5) | 180,825  (42.1) | 183,231  (41,4) | 180,451  (41.0) | 176,299  (40.7) | 165,720  (40.1) | 154,462  (39.0) | 143,375  (38.2) | 139,428  (38.7) | -16.3 | *0.02* |
|  | ≥ 80 years | 60,134  (15.6) | 64,216  (16.1) | 68,223  (16.6) | 71,049  (16.8) | 74,905  (17.4) | 80,389  (18.1) | 84,430  (19.2) | 84,690  (19.5) | 84,119  (20.4) | 82,865  (20.9) | 76,571  (20.4) | 77,322  (21.5) | +28.6 | *0.004* |
| male ICU  admissions  /  100.000  inhabitants | general  population | 985.9 | 1012.0 | 1027.8 | 1065.1 | 1069.3 | 1091.1 | 1078.9 | 1059.8 | 1007.2 | 964.8 | 913.2 | 886.1 | -10.1 | 0.08 |
|  | < 65 years | 496.3 | 505.5 | 512.4 | 531.8 | 531.2 | 543.1 | 528.7 | 520.7 | 492.4 | 479.7 | 469.7 | 440.4 | -11.3 | *0.04* |
|  | 65-79 years | 2,931.4 | 3,007.7 | 3,042.7 | 3,107.0 | 3,113.7 | 3,148.7 | 3,100.0 | 3,035.4 | 2,867.4 | 2,685.6 | 2,497.5 | 2,417.1 | -17.5 | *0.008* |
|  | ≥ 80 years | 4,396.9 | 4,514.5 | 4,647.2 | 4,620.1 | 4,575.6 | 4,613.8 | 4,561.0 | 4,309.7 | 4,016.3 | 3,726.7 | 3,293.5 | 3,351.5 | -23.7 | *0.001* |
| female ICU  admissions  [total numbers  (%)] | general  population | 281,213  (100) | 288,737  (100) | 295,662  (100) | 304,259  (100) | 306,861  (100) | 317,336  (100) | 313,841  (100) | 305,874  (100) | 288,958  (100) | 270,732  (100) | 250,410  (100) | 243,565  (100) | -13.4 | *0.07* |
|  | < 65 years | 89,545  (31.8) | 92,281  (32.0) | 95,260  (32.2) | 97,605  (32.1) | 99,033  (32.3) | 102,423  (32.3) | 100,495  (32.0) | 99,204  (32.4) | 93,762  (32.4) | 88,714  (32.8) | 85,215  (34.0) | 80,845  (33.2) | -9.7 | 0.16 |
|  | 65-79 years | 111,160  (39.5) | 113,840  (39.4) | 116,503  (39.4) | 119,896  (39.4) | 119,463  (38.9) | 122,334  (38.6) | 119,762  (38.2) | 115,112  (37.6) | 107,201  (37.1) | 98,488  (36.4) | 90,442  (36.1) | 87,768  (36.0) | -21.0 | *0.009* |
|  | ≥ 80 years | 80,508  (28.6) | 82,616  (28.6) | 83,899  (28.4) | 86,758  (28.5) | 88,365  (28.8) | 92,579  (29.2) | 93,584  (29.8) | 91,558  (29.9) | 87,995  (30.5) | 83,530  (30.9) | 74,753  (29.9) | 74,952  (30.8) | -6.9 | 0.46 |
| female ICU  admissions  /  100.000  inhabitants | general  population | 684.3 | 701.8 | 715.1 | 736.9 | 739.2 | 760.2 | 749.3 | 728.3 | 686.5 | 642.6 | 594.1 | 581.6 | -15.0 | *0.03* |
|  | < 65 years | 283.7 | 292.1 | 299.9 | 308.6 | 311.8 | 321.1 | 314.5 | 310.2 | 293.2 | 277.9 | 267.5 | 256.0 | -9.8 | 0.11 |
|  | 65-79 years | 1,664.4 | 1,706.8 | 1,737.9 | 1,779.8 | 1,769.7 | 1,812.3 | 1,776.3 | 1,713.3 | 1,606.6 | 1,487.6 | 1,372.6 | 1,332.9 | -19.9 | *0.007* |
|  | ≥ 80 years | 2,816.5 | 2,873.6 | 2,915.2 | 2,973.7 | 2,945.8 | 2,993.0 | 2,928.9 | 2,770.5 | 2,557.5 | 2,329.8 | 2,020.8 | 2,016.0 | -28.5 | *0.002* |

| **sex and age-specific deaths in German ICUs** | | | | | | | | | | | | | | | |
| --- | --- | --- | --- | --- | --- | --- | --- | --- | --- | --- | --- | --- | --- | --- | --- |
| male ICU  deaths  [total numbers  (%)] | general  population | 54,468  (100) | 56,092  (100) | 59,299  (100) | 59,244  (100) | 63,137  (100) | 64,637  (100) | 66,168  (100) | 66,867  (100) | 65,374  (100) | 68,007  (100) | 74,452  (100) | 68,799  (100) | +26.3 | *<0.001* |
|  | < 65 years | 13,468  (27.4) | 13,497  (24.1) | 14,043  (23.7) | 14,133  (23.9) | 14,848  (23.5) | 15,406  (23.8) | 14,960  (22.6) | 15,643  (23.4) | 14,959  (22.9) | 15,768  (23.2) | 18,421  (24.7) | 16,266  (23.6) | +20.8 | *<0.001* |
|  | 65-79 years | 26,279  (48.2) | 26,782  (47.7) | 28,040  (47.3) | 27,952  (47.2) | 29,397  (46.6) | 29,409  (45.5) | 29,849  (45.1) | 29,521  (44.1) | 28,504  (43.6) | 28,962  (42.6) | 32,072  (43.1) | 28,714  (41.7) | +9.3 | *0.01* |
|  | ≥ 80 years | 14,721  (27.0) | 15,813  (28.2) | 17,216  (29.0) | 17,159  (29.0) | 18,892  (29.9) | 19,822  (30.7) | 21,359  (32.3) | 21,703  (32.5) | 21,911  (33.5) | 23,277  (34.2) | 23,959  (32.2) | 23,819  (34.6) | +61.8 | *<0.001* |
| male ICU  deaths  /  100.000  inhabitants | general  population | 139.0 | 142.4 | 148.5 | 149.2 | 157.2 | 159.2 | 162.3 | 163.5 | 159.4 | 165.7 | 181.4 | 169.2 | -4.9 | *<0.001* |
|  | < 65 years | 41.9 | 41.8 | 42.9 | 43.6 | 45.4 | 46.6 | 45.2 | 47.2 | 45.2 | 47.7 | 55.9 | 49.9 | +21.7 | *<0.001* |
|  | 65-79 years | 462.2 | 470.5 | 489.0 | 483.7 | 506.2 | 505.4 | 512.8 | 508.3 | 493.2 | 503.5 | 558.7 | 497.8 | +19.1 | *0.01* |
|  | ≥ 80 years | 1,076.4 | 1,111.7 | 1,172.7 | 1,115.8 | 1,154.0 | 1,137.6 | 1,153.8 | 1,104.4 | 1,046.1 | 1,046.8 | 1,030.5 | 1,032.4 | +7.7 | *0.03* |
| male ICU  deaths  /  100 deaths  in Germany | all German  deaths | 13.4 | 13.5 | 13.8 | 14.0 | 14.0 | 14.4 | 14.5 | 14.2 | 14.0 | 13.8 | 14.4 | 12.9 | -3.7 | 0.78 |
|  | < 65 years | 15.0 | 15.1 | 15.5 | 15.9 | 16.2 | 17.0 | 16.9 | 17.3 | 17.1 | 17.6 | 19.5 | 17.6 | 17.3 | *<0.001* |
|  | 65-79 years | 16.1 | 16.3 | 16.7 | 17.1 | 17.3 | 17.7 | 18.0 | 17.8 | 17.8 | 17.9 | 19.3 | 17.1 | 6.2 | *0.005* |
|  | ≥ 80 years | 9.5 | 9.7 | 10.0 | 10.1 | 10.0 | 10.4 | 10.5 | 10.1 | 10.0 | 9.6 | 9.4 | 8.8 | -7.3 | 0.25 |
| female ICU  deaths  [total numbers  (%)] | general  population | 41,678  (100) | 41,832  (100) | 44,023  (100) | 43,786  (100) | 45,729  (100) | 46,425  (100) | 47,224  (100) | 46,933  (100) | 45,528  (100) | 45,007  (100) | 47,527  (100) | 45,441  (100) | +9.0 | *0.007* |
|  | < 65 years | 7,488  (18.0) | 7,308  (17.5) | 7,992  (18.2) | 7,906  (18.1) | 8,114  (17.7) | 8,392  (18.1) | 8,393  (17.8) | 8,707  (18.6) | 8,348  (18.3) | 8,230  (18.3) | 9,330  (19.6) | 8,877  (19.5) | +18.6 | *<0.001* |
|  | 65-79 years | 16,491  (39.6) | 16,355  (39.1) | 17,332  (39.4) | 17,380  (39.7) | 18,038  (39.4) | 18,272  (39.4) | 18,310  (38.8) | 18,255  (38.9) | 17,238  (37.9) | 17,114  (38.0) | 18,566  (39.1) | 17,180  (37.8) | +4.2 | 0.15 |
|  | ≥ 80 years | 17,699  (42.5) | 18,169  (43.4) | 18,699  (42.5) | 18,500  (42.3) | 19,577  (42.8) | 19,761  (42.6) | 20,521  (43.5) | 19,971  (42.6) | 19,942  (43.8) | 19,663  (43.7) | 19,631  (41.3) | 19,384  (42.7) | +9.5 | *0.009* |
| female ICU  deaths  /  100.000  inhabitants | general  population | 101.4 | 101.7 | 106.5 | 106.1 | 110.2 | 111.2 | 112.7 | 111.7 | 108.2 | 106.8 | 112.8 | 108.5 | +7.0 | *0.02* |
|  | < 65 years | 23.7 | 23.1 | 25.2 | 25.0 | 25.5 | 26.3 | 26.3 | 27.2 | 26.1 | 25.8 | 29.3 | 28.1 | +8.6 | *<0.001* |
|  | 65-79 years | 246.9 | 245.2 | 258.5 | 258.0 | 267.2 | 270.7 | 271.6 | 271.7 | 258.3 | 258.5 | 281.8 | 260.9 | +5.7 | *0.04* |
|  | ≥ 80 years | 619.2 | 632.0 | 649.7 | 634.1 | 652.6 | 638.9 | 642.2 | 604.3 | 579.6 | 548.4 | 530.7 | 521.4 | -15.8 | *0.001* |
| female ICU  deaths  /  100 deaths  in Germany | all German  deaths | 9.4 | 9.2 | 9.5 | 9.8 | 9.6 | 10.0 | 10.0 | 9.7 | 9.6 | 9.1 | 9.4 | 8.5 | -9.6 | 0.26 |
|  | < 65 years | 15.4 | 15.3 | 16.3 | 16.6 | 16.5 | 17.1 | 17.3 | 17.6 | 17.4 | 17.1 | 18.7 | 17.6 | 14.3 | *<0.001* |
|  | 65-79 years | 15.1 | 14.8 | 15.2 | 15.7 | 15.7 | 16.4 | 16.4 | 16.2 | 15.9 | 15.9 | 16.8 | 15.3 | 1.3 | 0.05 |
|  | ≥ 80 years | 6.2 | 6.2 | 6.2 | 6.4 | 6.3 | 6.5 | 6.5 | 6.2 | 6.3 | 5.8 | 5.6 | 5.2 | -16.1 | *0.02* |

ICU = intensive care unit

## Table 9. Incidence and mortality of organ replacement therapies in German ICUs (2011–2022)

| **organ replacement**  **therapies** | | | **2011** | **2012** | **2013** | **2014** | **2015** | **2016** | **2017** | **2018** | **2019** | **2020** | **2021** | **2022** | **relative**  **change**  **[%]** | ***p*-value** |
| --- | --- | --- | --- | --- | --- | --- | --- | --- | --- | --- | --- | --- | --- | --- | --- | --- |
| **VT ……** | total numbers | | 181,705 | 190,495 | 203,402 | 209,996 | 224,852 | 236,507 | 243,470 | 242,979 | 245,671 | 246,880 | 264,863 | 245,853 | +35.3 | *<0.001* |
|  | proportion of ICU  admissions [%] | | 27.2 | 27.7 | 28.2 | 28.5 | 30.5 | 31.1 | 32.3 | 32.9 | 35.0 | 37.0 | 42.4 | 40.7 | +49.6 | *<0.001* |
|  | < 65  years | total  numbers | 63,708 | 65,801 | 71,216 | 72,749 | 77,673 | 82,076 | 82,957 | 83,908 | 84,742 | 87,197 | 99,384 | 87,476 | +37.3 | *<0.001* |
|  |  | proportion of ICU  admissions [%] | 25.6 | 25.8 | 27.1 | 27.0 | 28.5 | 29.1 | 30.1 | 30.9 | 33.0 | 35.3 | 41.4 | 39.0 | +52.4 | *<0.001* |
|  |  | mortality  [%] | 21.4 | 20.5 | 20.2 | 19.8 | 19.9 | 19.5 | 19.0 | 20.0 | 19.0 | 19.6 | 20.5 | 20.9 | -2.2 | 0.47 |
|  | 65-79  years | total  numbers | 81,712 | 85,593 | 90,580 | 94,227 | 100,032 | 103,791 | 105,951 | 104,638 | 104,045 | 102,527 | 107,397 | 100,690 | +23.2 | *<0.001* |
|  |  | proportion of ICU  admissions [%] | 29.4 | 30.0 | 31.1 | 31.5 | 33.3 | 34.0 | 35.3 | 35.9 | 38.1 | 40.5 | 45.9 | 44.3 | +50.7 | *<0.001* |
|  |  | mortality  [%] | 33.1 | 32.1 | 32.2 | 31.1 | 31.4 | 30.6 | 30.5 | 30.9 | 30.3 | 32.1 | 35.5 | 33.3 | +0.7 | 0.48 |
|  | ≥ 80  years | total  numbers | 36,285 | 39,101 | 41,606 | 43,020 | 47,147 | 50,640 | 54,562 | 54,433 | 56,884 | 57,156 | 58,082 | 36,285 | +59.0 | *<0.001* |
|  |  | proportion of ICU  admissions [%] | 25.8 | 26.6 | 27.4 | 27.3 | 28.9 | 29.3 | 30.7 | 30.9 | 33.1 | 34.4 | 38.4 | 37.9 | +46.8 | *<0.001* |
| **RRT** | total numbers | | 54,561 | 55,469 | 56,171 | 56,697 | 58,987 | 61,944 | 61,937 | 62,267 | 61,558 | 62,758 | 65,065 | 59,024 | +8.2 | *0.002* |
|  | proportion of ICU  admissions [%] | | 8.2 | 8.1 | 7.8 | 7.7 | 8.0 | 8.2 | 8.2 | 8.4 | 8.8 | 9.4 | 10.4 | 9.8 | +19.7 | *<0.001* |
|  | < 65  years | total  numbers | 17,678 | 17,288 | 18,446 | 18,509 | 19,505 | 20,642 | 20,418 | 21,141 | 20,780 | 21,566 | 23,458 | 20,932 | +18.4 | *<0.001* |
|  |  | proportion of ICU  admissions [%] | 7.1 | 6.8 | 7.0 | 6.9 | 7.2 | 7.3 | 7.4 | 7.8 | 8.1 | 8.7 | 9.8 | 9.3 | +31.4 | *<0.001* |
|  |  | mortality  [%] | 38.2 | 38.3 | 39.2 | 39.5 | 39.7 | 39.1 | 39.2 | 39.8 | 38.9 | 39.8 | 44.6 | 40.9 | +7.0 | *0.02* |
|  | 65-79  years | total  numbers | 26,822 | 27,864 | 27,375 | 27,682 | 28,410 | 29,497 | 29,055 | 28,746 | 28,128 | 28,059 | 28,740 | 25,817 | −3.8 | 0.87 |
|  |  | proportion of ICU  admissions [%] | 9.7 | 9.8 | 9.4 | 9.2 | 9.5 | 9.7 | 9.7 | 9.9 | 10.3 | 11.1 | 12.3 | 11.4 | +17.7 | *0.002* |
|  |  | mortality  [%] | 46.1 | 45.4 | 46.6 | 46.1 | 47.5 | 47.0 | 48.1 | 47.7 | 47.8 | 49.7 | 52.8 | 50.2 | +9.0 | *<0.001* |
|  | ≥ 80  years | total  numbers | 10,061 | 10,317 | 10,350 | 10,506 | 11,072 | 11,805 | 12,464 | 12,380 | 12,650 | 13,133 | 12,867 | 12,275 | +22.0 | *<0.001* |
|  |  | proportion of ICU  admissions [%] | 7.2 | 7.0 | 6.8 | 6.7 | 6.8 | 6.8 | 7.0 | 7.0 | 7.4 | 7.9 | 8.5 | 8.1 | +12.7 | *0.004* |
|  |  | mortality  [%] | 50.9 | 51.7 | 52.5 | 51.4 | 53.6 | 53.0 | 52.9 | 53.6 | 52.7 | 55.2 | 56.4 | 55.5 | +9.2 | *0.008* |
| **ECMO** | total numbers | | 2,461 | 2,723 | 4,014 | 4,477 | 5,249 | 5,856 | 6,256 | 6,733 | 7,045 | 7,524 | 10,242 | 7,000 | +184.4 | <0.001 |
|  | proportion of ICU  admissions [%] | | 0.4 | 0.4 | 0.6 | 0.6 | 0.7 | 0.8 | 0.8 | 0.9 | 1.0 | 1.1 | 1.6 | 1.2 | +213.5 | *<0.001* |
|  | < 65  years | total  numbers | 1,649 | 1,731 | 2,601 | 2,566 | 3,043 | 3,449 | 3,571 | 3,871 | 3,961 | 4,497 | 7,026 | 4,400 | +166.8 | *<0.001* |
|  |  | proportion of ICU  admissions [%] | 0.7 | 0.7 | 1.0 | 1.0 | 1.1 | 1.2 | 1.3 | 1.4 | 1.5 | 1.8 | 2.9 | 2.0 | +197.0 | *<0.001* |
|  |  | mortality  [%] | 52.8 | 55.1 | 50.5 | 50.9 | 50.8 | 47.7 | 47.3 | 48.7 | 47.8 | 50.4 | 54.2 | 50.1 | -5.1 | 0.34 |
|  | 65-79  years | total  numbers | 728 | 900 | 1,180 | 1,528 | 1,802 | 1,937 | 2,161 | 2,300 | 2,410 | 2,531 | 2,751 | 2,119 | +191.2 | *<0.001* |
|  |  | proportion of ICU  admissions [%] | 0.3 | 0.3 | 0.4 | 0.5 | 0.6 | 0.6 | 0.7 | 0.8 | 0.9 | 1.0 | 1.2 | 0.9 | +257.7 | *<0.001* |
|  |  | mortality  [%] | 74.0 | 71.4 | 71.1 | 62.3 | 62.4 | 58.8 | 57.5 | 58.8 | 58.1 | 60.2 | 64.5 | 59.6 | -19.5 | *<0.001* |
|  | ≥ 80  years | total  numbers | 84 | 92 | 233 | 383 | 404 | 470 | 524 | 562 | 674 | 496 | 465 | 481 | +472.6 | *0.002* |
|  |  | proportion of ICU  admissions [%] | 0.1 | 0.1 | 0.2 | 0.2 | 0.3 | 0.3 | 0.3 | 0.3 | 0.4 | 0.3 | 0.3 | 0.3 | +433.3 | *<0.001* |
|  |  | mortality  [%] | 72.6 | 56.5 | 36.5 | 32.4 | 31.7 | 31.7 | 27.9 | 32.7 | 27.2 | 32.1 | 33.1 | 29.1 | -59.9% | *0.009* |

ICU = intensive care unit, VT = mechanical ventilation, RRT = renal replacement therapy, ECMO = extracorporeal membrane oxygenation

## Figure 4. Incidence and mortality of organ replacement therapies in German ICUs (average across the observation period 2011–2022)


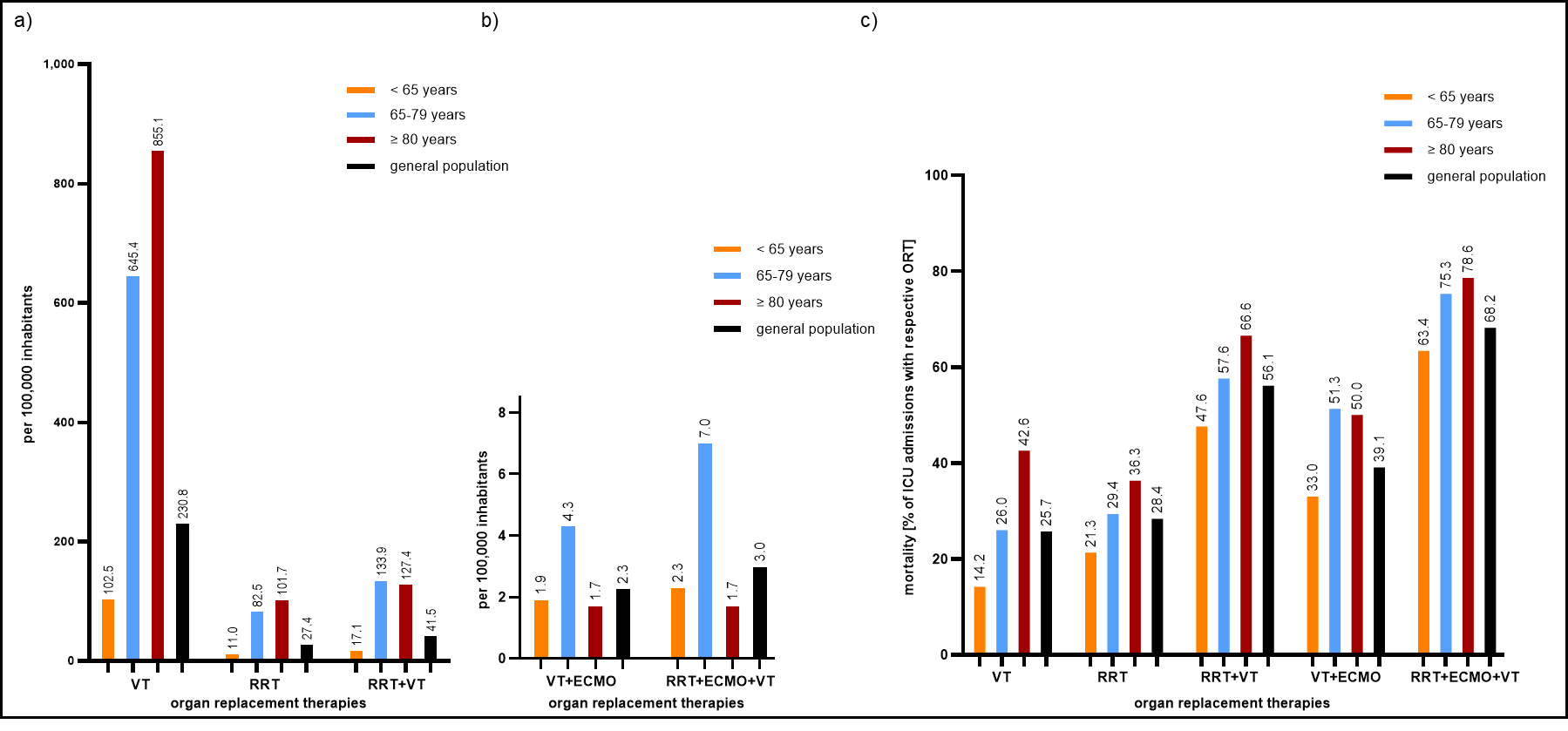


*The panels a) and b) show the incidence of organ replacement therapies (ORTs) per 100,000 inhabitants, for mechanical ventilation (VT) and renal replacement therapy (RRT) alone, as well as combination of different ORTs, for all patients and stratified by age group. The panel c) displays the mortality associated with single and combined ORTs for all patients and stratified by age group. Data are averages across the observation period 2011–2022. ECMO = extracorporeal membrane oxygenation. Significance levels: * = p < 0.05; ** = p < 0.01; *** = p < 0.001; n.s. = not significant (p ≥ 0.05).*

## Table 10. Use of single and combined organ replacement therapies in German ICUs (absolute numbers) (2011–2022)

|  | | **2011** | **2012** | **2013** | **2014** | **2015** | **2016** | **2017** | **2018** | **2019** | **2020** | **2021** | **2022** | **relative  change  [%]** | ***p*-value** |
| --- | --- | --- | --- | --- | --- | --- | --- | --- | --- | --- | --- | --- | --- | --- | --- |
| **only VT**  *(excl. RRT and ECMO)* | < 65  years | 53,058 | 55,068 | 59,386 | 61,028 | 64,806 | 68,232 | 69,193 | 69,634 | 70,072 | 71,334 | 80,538 | 71,628 | +35.0 | *<0.001* |
|  | 65-79  years | 66,035 | 69,068 | 74,031 | 77,481 | 82,152 | 85,141 | 87,162 | 86,117 | 85,318 | 82,975 | 86,408 | 82,380 | +24.8 | *0.002* |
|  | ≥ 80  years | 31,001 | 33,667 | 36,054 | 37,376 | 41,079 | 43,955 | 47,489 | 47,436 | 49,532 | 49,375 | 50,070 | 50,358 | +62.4 | *<0.001* |
| **only RRT**  *(excl. VT and ECMO)* | < 65  years | 7,397 | 6,926 | 7,181 | 7,293 | 7,348 | 7,563 | 7,423 | 7,575 | 7,077 | 6,734 | 6,501 | 6,256 | -15.4 | *0.04* |
|  | 65-79  years | 11,267 | 11,484 | 11,024 | 11,211 | 10,884 | 11,193 | 10,589 | 10,567 | 9,808 | 9,007 | 8,287 | 8,004 | -29.0 | *<0.001* |
|  | ≥ 80  years | 4,813 | 4,908 | 4,850 | 4,922 | 5,098 | 5,191 | 5,447 | 5,457 | 5,390 | 5,452 | 4,963 | 5,060 | +5.1 | 0.59 |
| **only RRT+VT**  *(excl.ECMO)* | < 65  years | 9,383 | 9,395 | 9,913 | 9,849 | 10,558 | 11,197 | 11,085 | 11,407 | 11,560 | 12,292 | 13,303 | 12,324 | +31.3 | *<0.001* |
|  | 65-79  years | 15,089 | 15,809 | 15,639 | 15,635 | 16,514 | 17,228 | 17,253 | 16,880 | 16,972 | 17,605 | 18,854 | 16,612 | +10.1 | *0.002* |
|  | ≥ 80  years | 5,216 | 5,366 | 5,435 | 5,484 | 5,879 | 6,495 | 6,895 | 6,774 | 7,113 | 7,560 | 7,786 | 7,097 | +36.1 | *<0.001* |
| **only**  **VT+ECMO**  *(excl. RRT)* | < 65  years | 560 | 575 | 904 | 848 | 1,058 | 1,166 | 1,217 | 1,217 | 1,440 | 1,532 | 2,684 | 1,601 | +185.9 | *<0.001* |
|  | 65-79  years | 197 | 250 | 336 | 439 | 532 | 534 | 562 | 610 | 656 | 743 | 831 | 675 | +242.6 | *<0.001* |
|  | ≥ 80  years | 39 | 27 | 64 | 83 | 107 | 93 | 71 | 102 | 114 | 114 | 121 | 122 | +212.8 | *<0.001* |
| **RRT+**  **ECMO+**  **VT** | < 65  years | 707 | 763 | 1,013 | 1,024 | 1,251 | 1,481 | 1,462 | 1,650 | 1,670 | 2,039 | 2,859 | 1,923 | +172.0 | *<0.001* |
|  | 65-79  years | 391 | 466 | 574 | 672 | 834 | 888 | 974 | 1,031 | 1,099 | 1,204 | 1,304 | 1,023 | +161.6 | *<0.001* |
|  | ≥ 80  years | 29 | 41 | 53 | 77 | 82 | 97 | 107 | 121 | 125 | 107 | 105 | 110 | +279.3 | *<0.001* |

## Table 11. Odds ratios for death with use of single and combined organ replacement therapies (2011–2022)

|  | | | **univariable logistic regression** | | | **multivariable logistic regression** | | |
| --- | --- | --- | --- | --- | --- | --- | --- | --- |
| **characteristic** | n | event n | OR | 95% CI | *p*-value | OR | 95% CI | *p*-value |
| age | 8,357,412 |  |  |  | <0.001 |  | | |
| < 65 years |  | 275,316 |  |  |  |  |  |  |
| 65-79 years |  | 553,366 | 2.04 | 2.03, 2.05 |  | 2.01 | 2.00, 2.02 | <0.001 |
| ≥ 80 years |  | 470,960 | 3.32 | 3.30, 3.33 |  | 4.13 | 4.11, 4.16 | <0.001 |
| **organ replacement therapy** | 8,357,412 |  |  | | <0.001 |  | | |
| none |  | 380,823 |  |  |  |  |  |  |
| VT |  | 583,747 | 4.50 | 4.48, 4.52 |  | 4.72 | 4.69, 4.74 | <0.001 |
| RRT |  | 76,850 | 5.19 | 5.14, 5.24 |  | 5.36 | 5.31, 5.41 | <0.001 |
| RRT+VT |  | 229,518 | 16.6 | 16.5, 16.8 |  | 19.1 | 19.0, 19.3 | <0.001 |
| VT+ECMO |  | 8,679 | 8.36 | 8.14, 8.59 |  | 13.1 | 12.8, 13.5 | <0.001 |
| RRT+VT+ECMO |  | 20,025 | 28.0 | 27.3, 28.7 |  | 44.5 | 43.4, 45.6 | <0.001 |

VT = mechanical ventilation, RRT = renal replacement therapy, ECMO = extracorporeal membrane oxygenation
